# Understanding the electrochemistry of “water-in-salt” electrolytes: basal plane highly ordered pyrolytic graphite as a model system†

**DOI:** 10.1039/d0sc01754j

**Published:** 2020-06-08

**Authors:** Pawin Iamprasertkun, Andinet Ejigu, Robert A. W. Dryfe

**Affiliations:** Department of Chemistry, University of Manchester Oxford Road Manchester M13 9PL UK robert.dryfe@manchester.ac.uk +44 (0)161-275-4598; National Graphene Institute, University of Manchester Oxford Road M13 9PL UK

## Abstract

A new approach to expand the accessible voltage window of electrochemical energy storage systems, based on so-called “water-in-salt” electrolytes, has been expounded recently. Although studies of transport in concentrated electrolytes date back over several decades, the recent demonstration that concentrated aqueous electrolyte systems can be used in the lithium ion battery context has rekindled interest in the electrochemical properties of highly concentrated aqueous electrolytes. The original aqueous lithium ion battery conception was based on the use of concentrated solutions of lithium bis(trifluoromethanesulfonyl)imide, although these electrolytes still possess some drawbacks including cost, toxicity, and safety. In this work we describe the electrochemical behavior of a simple 1 : 1 electrolyte based on highly concentrated aqueous solutions of potassium fluoride (KF). Highly ordered pyrolytic graphite (HOPG) is used as well-defined model carbon to study the electrochemical properties of the electrolyte, as well as its basal plane capacitance, from a microscopic perspective: the KF electrolyte exhibits an unusually wide potential window (up to 2.6 V). The faradaic response on HOPG is also reported using K_3_Fe(CN)_6_ as a model redox probe: the highly concentrated electrolyte provides good electrochemical reversibility and protects the HOPG surface from adsorption of contaminants. Moreover, this electrolyte was applied to symmetrical supercapacitors (using graphene and activated carbon as active materials) in order to quantify its performance in energy storage applications. It is found that the activated carbon and graphene supercapacitors demonstrate high gravimetric capacitance (221 F g^−1^ for activated carbon, and 56 F g^−1^ for graphene), a stable working voltage window of 2.0 V, which is significantly higher than the usual range of water-based capacitors, and excellent stability over 10 000 cycles. These results provide fundamental insight into the wider applicability of highly concentrated electrolytes, which should enable their application in future of energy storage technologies.

## Abstract 2

The stability of water-in-salt electrolyte systems is investigated using highly concentrated solutions of KF(aq) with graphite as a model system.

## Graphical abstract

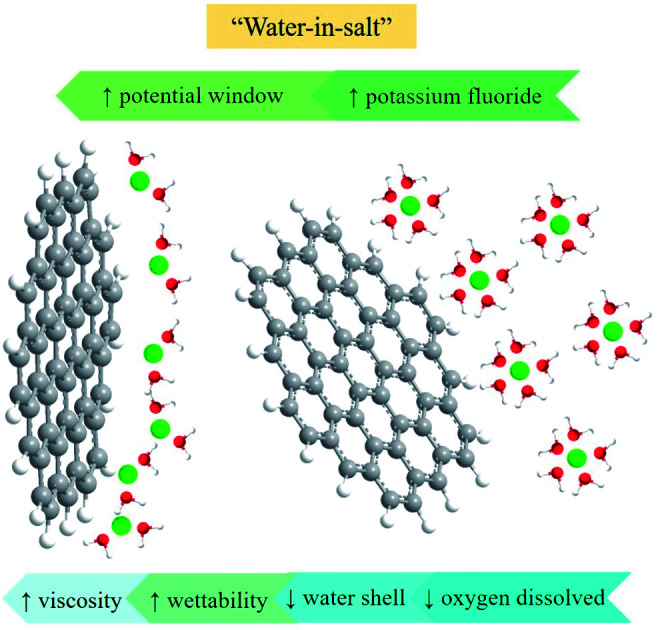

## Introduction

Carbon-based electrodes are central to most electrochemical energy storage and conversion applications (supercapacitors,^1^ fuel cells,^2^ redox flow batteries^3^). The anodes of lithium ion batteries are formed from graphite, conventionally coupled with aprotic electrolyte solutions, however recent reports have shown that reversible lithium ion cells can be based on aqueous electrolytes, if highly concentrated salts of Li^+^ with organic anions are employed.^4,5^ This approach has also been extended to supercapacitors, where an increased voltage window is also normally contingent on the use of organic electrolytes.^6^ These findings raise questions about the nature of the electrode/electrolyte interface, particularly the role of the solid electrolyte interface (SEI), and of the transport mechanisms operative in such highly concentrated electrolyte solutions. Another recurring question in electrochemical literature has been the understanding of the capacitance of graphite electrodes,^7–10^ indeed the role of water (and aqueous electrolytes) in protecting graphitic surfaces from ambient contamination has also been a recent focus of research interest.^11,12^

Binary electrolytes based on aqueous solutions of alkali metal halides are often studied as “model” electrolyte systems.^13^ Some of these electrolytes possess high solubility products (solubility exceeding 10 M), in particular alkali metal fluoride electrolytes have attracted particular interest in the electrochemical context.^14^ Potassium fluoride displays the highest solubility of the alkali metal halides (17 M at room temperature), although to the best of our knowledge there are no prior studies of electrochemical behavior of graphitic electrodes in such highly concentrated electrolytes. In this work, highly-oriented pyrolytic graphite (HOPG) was used as a well-defined model carbon material to study faradaic and non-faradaic electrochemistry in highly concentrated potassium fluoride, as a model “water-in-salt” electrolyte. The KF electrolyte exhibits a wide potential window, of 2.6 V, and the basal plane capacitance dependence on potential is reported. Transport in the electrolyte is correlated with its macroscopic viscosity. The work assists the development of a fuller understanding of the properties of “water-in-salt” systems and the associated optimisation of carbonaceous electrodes (*i.e.*, graphite, graphene and related materials) for high performance energy storage applications.

## Results and discussion

### Electrochemistry of HOPG in “water-in-salt” electrolyte

In order to study the properties of highly concentrated potassium fluoride (KF) aqueous electrolytes as a new water-in-salt electrolyte, varying concentrations of aqueous KF solutions were investigated in the three electrode system shown in Fig. S1.† Due to the high solubility of KF in water, electrolyte solutions of up to 17 M (25.7 m) can be prepared at room temperature (see Fig. S2†), markedly changing the association between the ions and the solvent, and hence the electrochemical (and other) properties of the electrolytes. Prior molecular dynamics simulations showed that, in LiTFSI water-in-salt electrolyte, the combination of high concentration of cation and fluorinated anion in water alters the hydration of the ions where the cation is strongly solvated, but the anion is not. The less solvated fluorinated anions form a percolating network that reduces the cation–anion pairs which enhances the transport number of the cations, compared to traditional dilute electrolytes.^15^ The structure of such water-in-salt systems is best described by the presence of highly solvated cations with 3D percolating channels for fast cation transport; and a less solvated aggregates of an anionic network with slow relaxation time that immobilises the anion movement.^16^ There have been some reports, *e.g.* molecular dynamics combined with neutron and X-ray diffraction, on the elucidation of structure in highly concentrated alkali metal fluoride solutions of the type used here,^17^ which show extensive ion pairing as would be predicted from classical electrochemical measurements.^13^ We note, however, that the structure of such concentrated electrolytes, both in the bulk and when confined, is a topic of current interest.^18^ The highly concentrated KF electrolyte therefore has a diminished water shell around its constituent ions, given that the mole fraction of the KF in the solution is approximately 0.32, this indicates that one hydrated ion pair is found for an average of 2.15 water molecules. This number is much lower than conventional electrolyte solutions,^19^ of 1 M concentration or less, which have excess water molecules in the system (mole fraction of water >0.98). For comparison, in dilute electrolyte systems, the hydration number of normal aqueous system is reported to be about 5 to 6 molecules of water.^20–22^

**Fig. 1:**
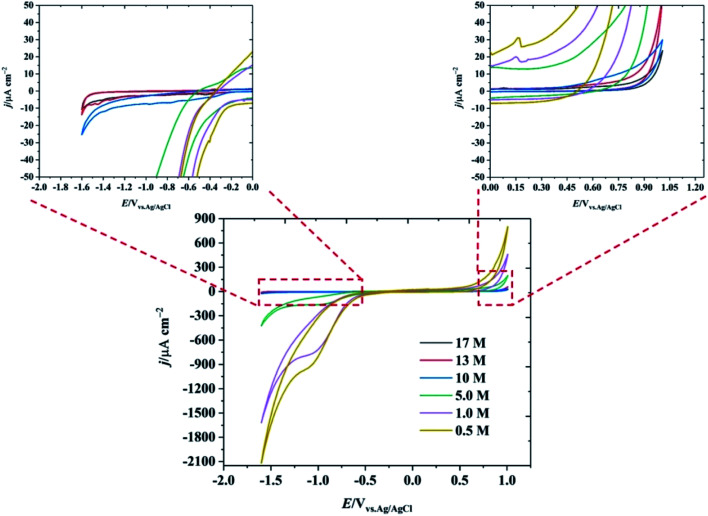
CV of basal plane highly ordered pyrolytic graphite at 1 V s^−1^ as a function of KF concentration. The insets show the magnified response at the extremes of the potential range.

The electrochemical potential window of water is relatively narrow (1.23 V on a purely thermodynamic basis). Even on relatively inert electrodes such as carbon, the kinetics of the hydrogen evolution reaction (HER) are relatively facile, compared to the anodic limit defined by the oxygen evolution reaction. The suppression of HER at extreme negative potentials using the water-in-salt approach is essential to increasing the operable potential range of water. Fig. 1 shows the electrochemical behaviour of the HOPG electrode as a function of KF concentration. It is seen that the negative potential limit increased by ∼1.0 V when the concentration of KF increased from 0.5 M to 17 M (magnified versions of the CVs, enabling further evaluation of the negative potential limit using a threshold current density of 1 mA cm^−2^ are presented in Fig. S4†). In addition, a reduction peak at −1.0 V is observed in lower KF concentrations due to the presence of dissolved oxygen, which is suppressed when the KF concentration increased to 17 M due to the decrease in oxygen solubility at higher concentration.^23^ The electrochemical window is also extended at positive potentials as the KF concentration is increased: it is concluded that the positive limit of the potential window for the highly concentrated electrolytes is limited by graphite oxidation, rather than oxygen evolution, (see Fig. S5† for further voltammetric data on the concentration dependence of the potential limits).^24^

### Capacitance of HOPG in “water-in-salt” electrolytes

As an alternative to the definition of the working potential range in these electrolytes by the use of rather arbitrary current density values, we have evaluated the working potential range of the electrolytes in a more rigorous fashion by using electrical impedance spectra to determine the range of potentials where the response is purely capacitive (see Fig. S6† and Fig. S7† for Bode and Nyquist plots). The capacitance of the HOPG electrode is then found by determining when the phase of the Bode plot approaches −90° (Fig. S6†).^25^ Overall, the capacitance of HOPG can be considered in terms of three components: (1) Helmholtz capacitance (*C*_H_), *i.e.*, the “inner” layer capacitance, (2) diffuse layer capacitance (*C*_diff_), which comes from the Gouy–Chapman layer, and (3) space charge capacitance (*C*_sc_), which arises from the limited number of charge carriers within the graphite electrode.^10,26^ The treatment of HOPG capacitance can be simplified because the *C*_diff_ term can be neglected at high ionic strength conditions, *i.e.* for electrolyte concentrations over 0.1 M (The Gouy–Chapman model can be used to give an approximate calculation of the *C*_diff_ term for these systems. For monovalent ions at 0.1 M, the calculated capacitance is close to 100 μF cm^−2^ at the potential zero charge, hereafter the “PZC”, *i.e.* significantly higher than the *C*_H_, and *C*_sc_ terms).^27^ Hence, the total measured capacitance (*C*) can be approximated as:^24,26^1
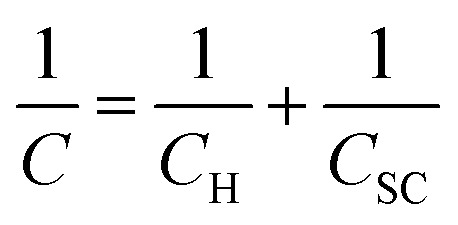


**Fig. 2:**
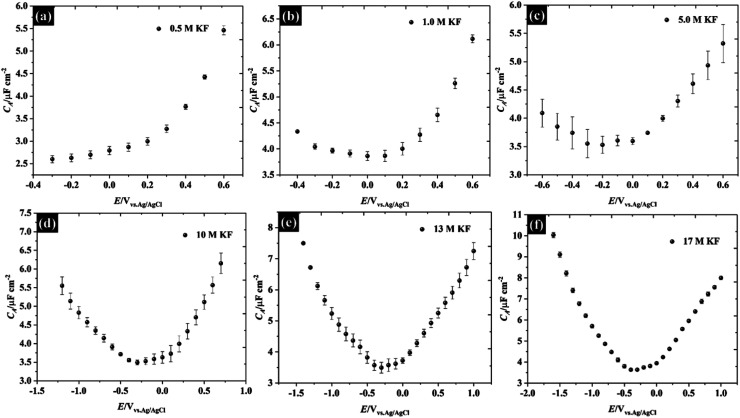
Capacitance–potential (*C*–*E*) curves of highly ordered pyrolytic graphite in (a) 0.5 M, (b) 1.0 M, (c) 5.0 M, (d) 10 M, (e) 13 M, and (f) 17 M potassium fluoride (water-in-KF salt).

Herein, the concentration of KF electrolytes was varied in the range 0.5–17 M. It can be clearly seen that the potential window found from the purely capacitive response of the KF electrolytes is enlarged when the concentration of KF increased, as can be seen in Fig. 2. The capacitance of HOPG in 0.5 M KF shows a minimum of 2.59 (±0.08) μF cm^−2^ but displays an asymmetry with respect to potential, due to the onset of an interfering faradaic process attributed to oxygen reduction at −0.3 V *vs.* Ag/AgCl.^28^ When the KF concentration was increased to 1.0 M, the *C*–*E* response became more symmetric showing a minimum capacitance of 3.86 (±0.10) μF cm^−2^ at 0.1 V *vs.* Ag/AgCl, which by definition is the potential zero charge (PZC) of the electrode/electrolyte interface.^7^ It can be seen that the capacitance of 1.0 M KF gradually increased on both positive and negative branches (from −0.4 V to 0.6 V). This suggests that the fluoride ions (*i.e.* at potentials positive of the PZC) are more strongly adsorbed on HOPG than potassium (at potentials negative of the PZC) at 1.0 M.^10^ Increasing the KF concentration, shifted the PZC to more negative potentials and extended the potential window to an unusually high value of 2.6 V (−1.6 to 1.0 V *vs.* Ag/AgCl at 17 M), which reflects a suppression of the water activity to an extent which increases the overpotential for water splitting.^29,30^ It is also concluded that without any applied potential (*i.e.* at the PZC), the potassium ions exhibit stronger adsorption properties on the basal plane than the fluoride ions, which is in good agreement with the DFT calculation of ions adsorption energy on graphene.^31^ The capacitance values at the PZC are summarised in Table 1. Overall, the capacitance at the PZC for all electrolyte concentrations is fairly consistent (∼3.5 μF cm^−2^), confirming the assumption made above that there is no contribution from diffuse layer capacitance.

**Table 1:** The potential of zero charge and capacitance of the HOPG in potassium fluoride electrolyte

Concentration of KF/M	PZC/V	*C*(*E* = PZC)/μF cm^−2^
0.5	—	Less than 2.59
1.0	0.1	3.86 ± 0.08
5.0	−0.2	3.52 ± 0.15
10	−0.3	3.49 ± 0.06
13	−0.3	3.49 ± 0.17
17	−0.4	3.63 ± 0.05

Also, the capacitances measured at the PZC for these electrolytes are in good agreement with previous reports for graphite *i.e.*, the minimum capacitances were reported to lie between 2.2 μF cm^−2^ and 4.8 μF cm^−2^ (depending on the electrolytes and measurement conditions).^7–10,26,32,33^ Note that all numerical capacitance data is summarised in Table S2.†

### Redox electrochemistry of HOPG

**Fig. 3:**
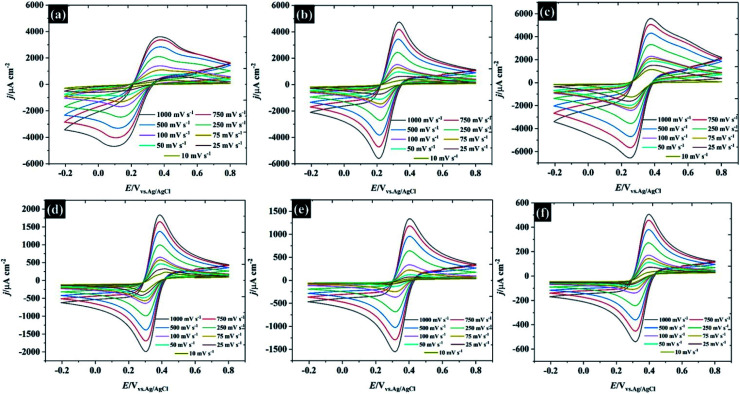
CVs recorded using 10 mM K_3_Fe(CN)_6_ in potassium fluoride as a supporting electrolyte on an HOPG working electrode. KF concentrations are: (a) 0.5 M, (b) 1.0 M, (c) 5.0 M, (d) 10 M, (e) 13 M, and (f) 17 M.

Following the analysis of capacitance, the redox electrochemistry of the prepared KF electrolytes was studied by adding 10 mM K_3_Fe(CN)_6_ to each of the KF solutions (see Fig. 3). Fig. 3a shows the CVs of K_3_Fe(CN)_6_ at HOPG using 0.5 M KF supporting electrolyte. The redox chemistry of K_3_Fe(CN)_6_ displays broad oxidation and reduction peaks with a wide peak-to-peak separation (Δ*E*_p_) that ranges between 220 and 292.5 mV depending on scan rate. The large Δ*E*_p_ value suggests that the kinetics of electron transfer are slow when using 0.5 M KF. However, the Δ*E*_p_ value significantly decreased as the KF concentration is increased to 5.0 M, suggesting the kinetics of electron transfer processes in these electrolytes becomes faster: for example the Δ*E*_p_ at a low scan rate (10 mV s^−1^) was reduced to 116.4 mV (for 1.0 M KF) and 100.6 mV (for 5.0 M KF). The plot of Δ*E*_p_ as a function of scan rate for each of the KF concentrations can be found in Fig. S8.† Interestingly, the electron transfer process of the K_3_Fe(CN)_6_ apparently became more reversible when the electrolyte reached the “water-in-salt” regime (at 10 M) showing a Δ*E*_p_ of about 81 mV for most of the scan rates employed. However, the observed Δ*E*_p_ for this reaction is still higher than the ideal value of 59 mV, expected for an electrochemically reversible one electron transfer process.^27^ Note also that a shoulder is visible in the CVs at lower KF concentrations (Fig. 3a–c), this phenomenon becomes more prominent on repeated scanning and is discussed in more detail below. A further observation is the positive shift in the equilibrium potential as the electrolyte concentration is increased. This is consistent with previous work by Peter *et al.*, who rationalised the shift in terms of the enhanced ion pairing between the electrode product (ferrocyanide) and the electrolyte cation, which changes the activity coefficient ratio and hence the formal potential.^39^

**Fig. 4:**
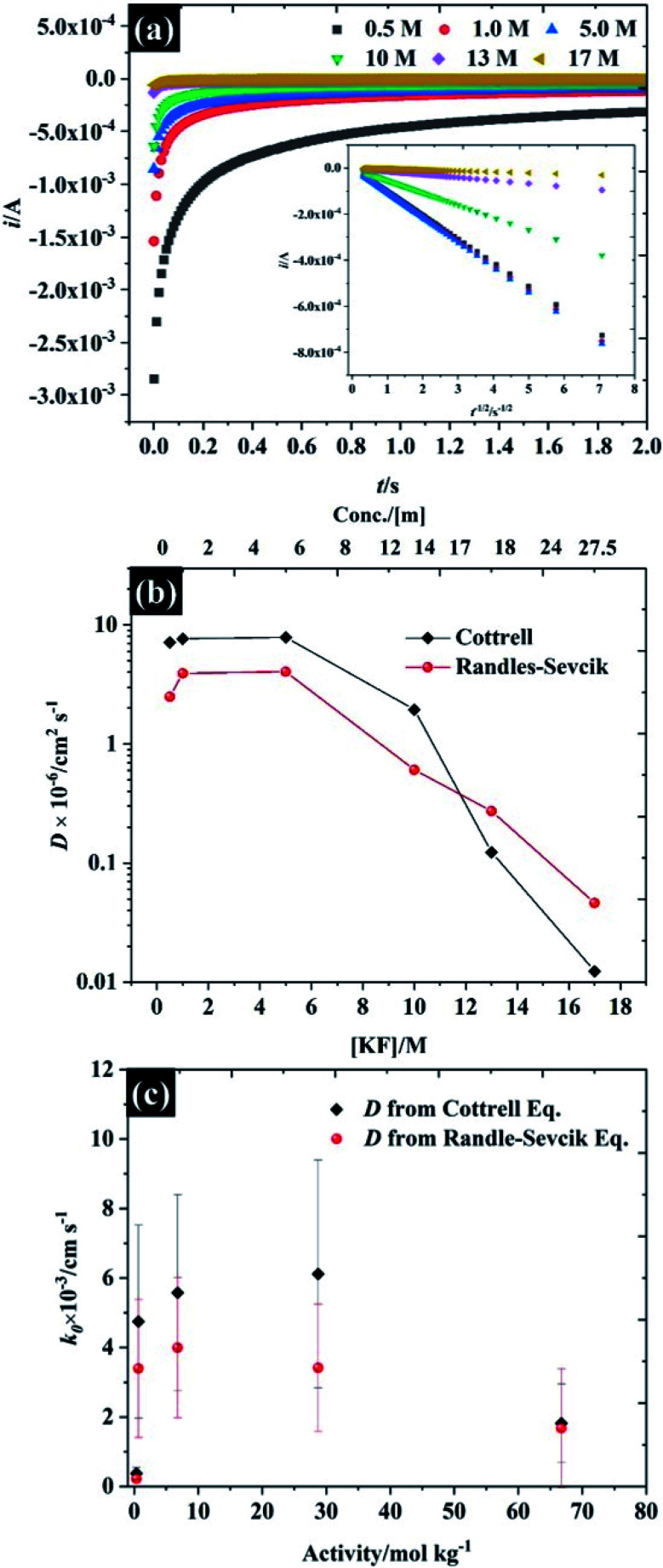
Investigation of the diffusion coefficient of the ferro/ferri cyanide redox spices using 10 mM K_3_Fe(CN)_6_ as a function of KF concentration (a) chronoamperometry, (b) diffusion coefficient, and (c) dependence of the heterogeneous electron transfer rate constant on electrolyte activity using reported KF activity coefficients.^40,41^ Note the semi-logarithmic scale of (b).

To further understand the electrochemical properties of these electrolytes, the chronoamperometric (CA) response at each KF concentration is presented in Fig. 4a. The current (*i*) of all electrolytes displays a linear dependence on the inverse square root of time (see inset figure) after the potential was stepped from a value where no faradaic reaction takes place to a potential in which the reduction reaction was controlled by the diffusion process. It is obvious that the gradient of these plots is significantly decreased with increased KF concentration, reflecting the higher viscosity of the electrolyte and hence the attenuated transport of K_3_Fe(CN)_6_. As the plot of *i vs. t*^−1/2^ (inset in Fig. 4a) is in excellent agreement with the Cottrell equation (eqn S(3)†), the diffusion coefficient (*D*) of the K_3_Fe(CN)_6_ can be calculated within each electrolyte solution as shown in Fig. 4b. Overall, it can be observed that the calculated *D* from the Cottrell equation is in reasonable agreement with the *D* from analysis of the CV data *via* the Randles–Sevčik equation.^27^ For the lower electrolyte concentrations *i.e.*, from 0.5 to 5.0 M, the calculated diffusion coefficients of K_3_Fe(CN)_6_ from both the CV and CA analyses lie between 2.49 × 10^−6^ and 7.87 × 10^−6^ cm^2^ s^−1^, which is in reasonable agreement with the value quoted for this solute in dilute aqueous solution.^34^ As the electrolyte starts to behave as a water-in-salt system, at 10 M, it is found that the diffusion coefficient of K_3_Fe(CN)_6_ decreased to *ca*. 1.94 × 10^−7^ cm^2^ s^−1^ and decreased further to 1.24 × 10^−8^ cm^2^ s^−1^ when the KF concentration was increased to 17 M. The latter values of diffusion coefficient are similar to those reported for related electroactive solutes in room temperature ionic liquids,^35^ reflecting the high viscosity of these water-in-salt electrolytes. To further explain the heterogeneous electron transfer kinetics of these electrolytes, the standard heterogeneous electron transfer rate constants (*k*_0_) were determined, using the dimensionless kinetic parameter (*ψ*) derived by Nicholson, from the dependence of peak separation on scan rate (see ESI†).^36,37^ It is evident that in 0.5 M KF (Fig. 4c) the average *k*_0_ is about 2 × 10^−4^ cm s^−1^ representing a quasi-reversible redox process, and in good agreement with previous literature.^38^ The *k*_0_ increased to *ca.* 4 × 10^−3^ cm s^−1^ when the concentration of KF was increased to 5.0 M, confirming the more reversible redox reaction at slightly elevated electrolyte concentrations. This behaviour is broadly consistent with an earlier report by Peter and co-workers on the effect of electrolyte on the kinetics of ferricyanide reduction on Au electrodes.^39^ A first-order dependence of electron transfer rate on potassium ion concentration was taken as evidence to support the operation of a “bridge” mechanism, where the reduced species exists as an ion pair with an alkali metal cation, and the electron transfer process proceeds *via* binding with a second cation. When the concentration is increased to 17 M, even though Δ*E*_p_ falls at these concentrations, the extraction of the kinetic parameter is convoluted with transport parameters because *ψ* also depends on the diffusion coefficient. The diffusion coefficient and *k*_0_ values for each of the electrolyte concentrations are given in Table S3.†

The fall in *k*_0_ observed at concentrations above 10 M (to *ca.* 1 × 10^−3^ cm s^−1^) is also consistent with the observations of Peter *et. al.*^39^ who found a deviation from the linear relation between *k*_0_ and electrolyte concentration at high concentrations when nitrate was used as the supporting electrolyte anion. This was attributed to the stronger binding of nitrate, relative to fluoride, to the alkali metal cation: the highest concentration in the earlier study was 10 M, hence it is likely that such a binding effect with fluoride is coming into play at the concentrations above 10 M.

### Viscosity of the electrolytes

**Fig. 5:**
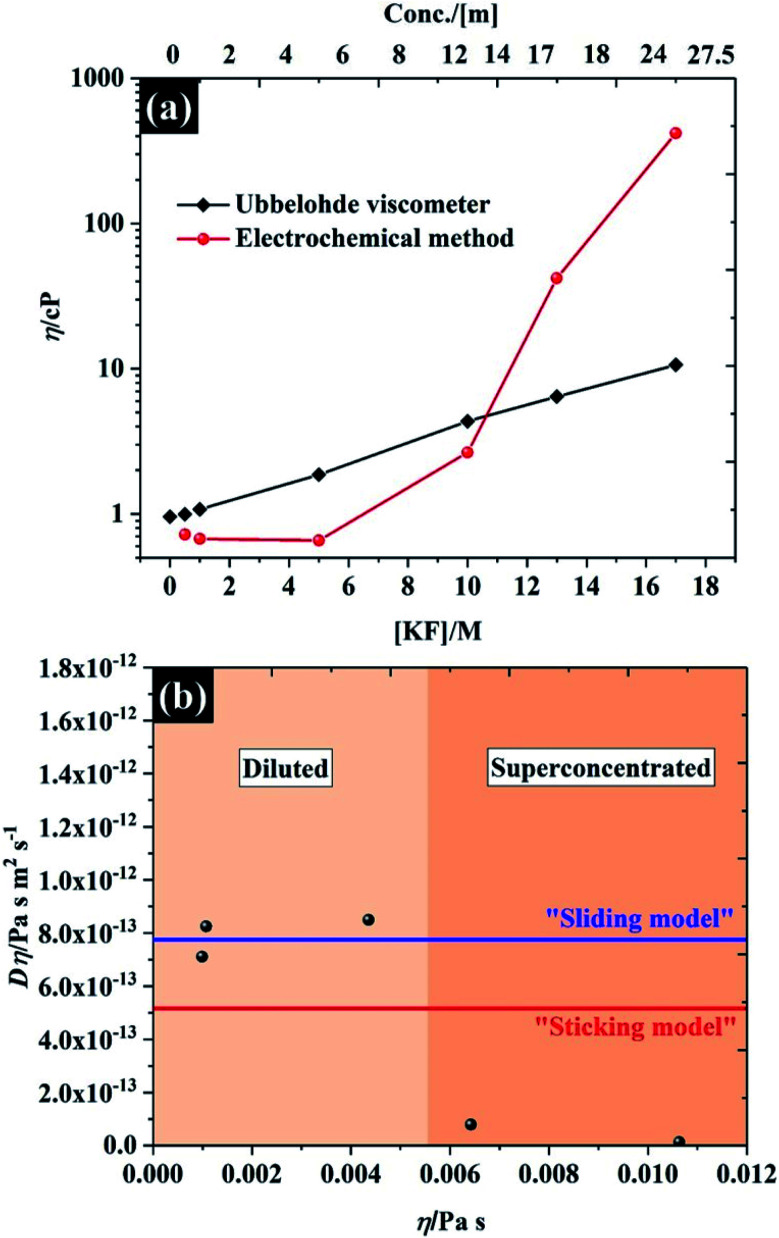
(a) viscosity of potassium fluoride electrolyte from Ubbelohde viscometry and the Stokes–Einstein equation applied to the chronoamperometric data, and (b) the product *D* × *η vs. η* for the KF data shown in (a). The upper (blue) line shows the predicted values determined using eqn (S8)† with the “Sutherland coefficient” of 4, and the lower (red) line shows the predicted values determined using eqn (S8)† with the numerical coefficient = 6. Each solid line was determined using a hydrodynamic radius of 0.422 nm for K_3_Fe(CN)_6_.^52^

The viscosity of the solutions, as a function of KF concentration in Fig. 5a, was compared focusing particularly on the comparison between macroscopic and microscopic measurements. The former were found using an Ubbelohde viscometer, which measures the kinematic viscosity (*v*) *via* the time for fluid flow (see Fig. S11† for numerical data on kinematic viscosity and density of the electrolytes). The microscopic analysis was derived from the electrochemical data reported above, where the Stokes–Einstein equation was used to obtain a viscosity from the observed solute diffusion coefficient (see eqn S(8)†).^35^ Overall, both methods give similar viscosity values, of between 0.65 cP and 1 cP at the low KF concentrations (below 5 M), which is comparable to the viscosity of pure water at 25 °C (∼0.00089 Pa *s* ≡ 0.89 cP)^42^ and agrees with the absolute viscosity (∼1 cP) of the aqueous electrolyte quoted in a previous report.^43^ It is clear that the viscosity of the electrolyte is very sensitive to the concentration of KF salt, as is reflected in the decreased self-diffusion coefficient of water reported for higher KF concentrations.^44,45^ Specifically the self-diffusion coefficient of water is reported to fall by a factor of 4.4, compared to pure water, when the electrolyte concentration is increased to 15.51 m (∼12 M),^44^ which agrees with our observation from the Ubblohde viscometer, that the viscosity of the 10 M solution is 4.3 cP. This exponential dependence of viscosity on 1 : 1 alkali metal halide electrolyte concentration was also reported by Abdulagatov *et. al*., who found that the viscosity of an aqueous NaI solution increased exponentially up to about 3 cP at the maximum concentration of ∼11 m.^46,47^ However for very high concentrations of electrolyte (>10 M), the Stokes–Einstein equation predicts a much higher viscosity attenuation than the measurements *via* the Ubblohde viscometer indicate, *i.e.* the viscosity increased by two orders of magnitude (to ∼417 cP, at 17 M KF). One explanation may be the basis of the Stokes–Einstein equation itself, as it does not apply perfectly to highly viscous electrolytes such as ionic liquids. As shown in Fig. 5b, for KF concentrations above 10 M, neither the “sliding sphere model” where the denominator reduces to 4π*ηr* or the “sticking sphere model” (where the denominator is 6π*ηr*) of the Stokes–Einstein equation fit the data satisfactorily. However, for KF concentrations below 10 M, the data fits the sliding sphere model.^35^ For electrolyte concentrations ≥10 M, there may be “sticking” between the ferricyanide species and constituent ions of KF. This hypothesis is also in good agreement with molecular dynamics simulations of the concentration dependence of the viscosity of aqueous solutions using the Stokes–Einstein relationship, reporting that the viscosity ats high salt concentrations can be increased by up to 2 orders of magnitude when compared to dilute solution.^48^ The higher salt concentration decreases the diffusivity of the water because a higher proportion of solvent is incorporated into the hydration shell, similarly the ion transport is decreased due to enhanced ion-paring effects at higher concentration.^48^ The higher sensitivity of the solute diffusion coefficient inferred from voltammetry to KF concentration could also indicate that a more complex process is inhibiting the flux of the solute. One possibility is that there is higher degree of ion pairing of the ferricyanide due to its high charge, as the rate constant dependence on concentration implies, and that a complex ion-paired structure is responsible for transport to the electrode at high KF concentrations. It is found that the viscosity of water-in-salt electrolyte (17 M KF) can approach those of ionic liquid electrolytes;^49,50^ thus, it can be concluded that the extended electrochemical potential window is related to the change in transport within the solution and hence solution viscosity,^44,45^ which reduced the oxygen transport properties in the electrolyte.^51^ Note, the numerical data of the viscosity from both techniques are presented in Table S4.†

### Effect of air and waterborne hydrocarbons

**Fig. 6:**
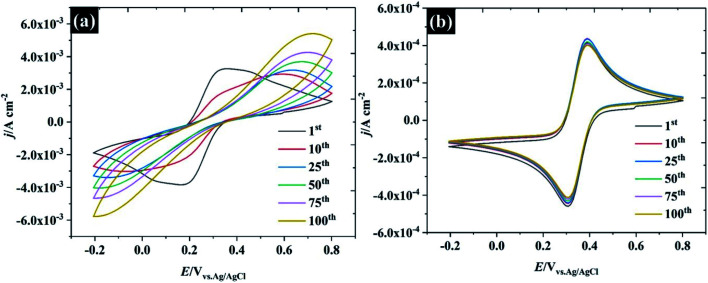
Repeated CV cycles at 100 mV s^−1^ at HOPG in 10 mM K_3_Fe(CN)_6_ using (a) 0.5 M KF, and (b) 17 M KF as supporting electrolytes.

To further describe the effect of sample history on the CV in water-in-salt electrolyte, consecutive CVs of HOPG were carried out in two different supporting electrolyte concentrations: (1) 0.5 M KF, and (2) 17 M KF as shown in Fig. 6. It is clear that only the first CV cycle of 0.5 M KF exhibits a broad oxidation/reduction peak for ferri/ferrocyanide, with Δ*E*_p_ about 220 mV, indicating that a quasi-reversible redox process occurs. On successive cycling, Δ*E*_p_ is increased with a significant change in wave shape indicating a further retardation of electron transfer, which is attributed to the passivation induced by decomposition of the ferri/ferro cyanide couple at the HOPG surface.^53^ Despite being widely used as a “model” redox couple, the electrochemical behaviour of ferri/ferrocyanide is, in fact, complex, as the preceding discussion on heterogeneous kinetics indicates. As well as the influence of ion-paired intermediates on the electrode process, a further consideration is the decomposition of the electrode reactants to form mixed valent Prussian Blue type complexes. As discussed in the context of electron transfer kinetics, there is evidence that the association of the electrolyte cation with ferrocyanide in particular can slow down the decomposition process according to eqn (2).^54^ This is consistent with the CV response obtained in 17 M KF, which is remarkably stable, suggesting that the concentrated KF can prevent the ferri/ferro cyanide-induced passivation of the HOPG surface.

2 Fe(CN)_6_^4−^ + K^+^ ↔ KFe(CN)_6_^3−^

**Fig. 7:**
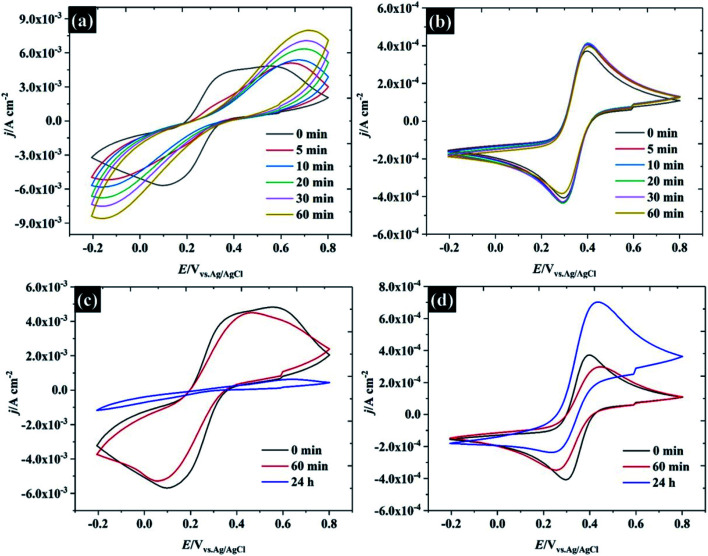
CV of HOPG for the reduction of 10 mM Fe(CN)_6_^3−^ in aged KF electrolyte at a fixed concentration of (a) 0.5 M, and (b) 17 M. CV for the reduction of 10 mM Fe(CN)_6_^3−^ in air aged at a fixed concentration of (c) 0.5 M, and (d) 17 M.

Several factors could drive the sensitivity of the voltammetric stability to electrolyte concentration. In particular, a detailed study by the group of Unwin^53^ has shown that reversible voltammetry can be obtained on the basal plane of freshly exfoliated HOPG surfaces, although the reversibility of the response rapidly degraded on successive cycling and/or if the HOPG were left to “age” in air. Importantly, other redox couples did not show such sensitivity to cycle number or ageing. Similar observations were made by Compton *et. al.* with basal and edge plane HOPG,^55^ although the initial response on the basal plane was not electrochemically reversible. To probe these effects in highly concentrated electrolytes, HOPG electrodes were aged under different conditions: (1) in air, (2) in electrolyte (see Fig. 7). For the ageing in the electrolyte (containing 10 mM K_3_Fe(CN)_6_ and the desired concentration of KF), the HOPG was cleaved with “scotch” tape, placed in the PTFE cell and solution introduced immediately. CVs were then recorded at *t* = 0 min and subsequently recorded at 5, 10, 20, 30, and 60 min. It was found, in agreement with the work of Unwin and co-workers, that the Δ*E*_p_ of 0.5 M KF (Fig. 7a) is significantly increased from *t* = 0 to *t* = 5 min,^53^ a change accompanied by an evolution in wave-shape even without any prior voltammetry being recorded. In contrast, the CV in Fig. 7b is relatively consistent, regardless of sample aging, showing a minor shift between Δ*E*_p_ at *t* = 0 and *t* = 5 min; however, it is hard to draw a conclusion that this effect is due only to passivation by the electroactive species. Basal plane graphite is known to be susceptible to effects due to the adsorption of airborne^26^ and waterborne hydrocarbon species from the environment:^11^ such effects have a strong influence on ferri/ferrocyanide voltammetry; therefore, the change in CV shape may be due to hydrocarbon adsorption. To further explore the effect of ambient hydrocarbons, the HOPG sample was aged in air for different periods of time (0 min, 60 min, and 24 hours). It is clear that the Δ*E*_p_ of both 0.5 M and 17 M KF increased slightly from the initial values after ageing in air for 60 min; however, the CV of 17 M KF displays a much lower Δ*E*_p_ when compared to 0.5 M solutions at the same time. After the HOPG was left in air for 24 hours, the CV of 0.5 M KF (Fig. 7c) was completely changed, with the faradaic process being almost completely suppressed. By contrast, the CV of 17 M KF after ageing in air for 24 hours displays a slight increase of Δ*E*_p_ from the response at 60 min. It can be seen that the redox process is asymmetric, showing an oxidation current which exceeds the reduction current; thus, it is concluded that the adsorbed hydrocarbon is involved in this reaction. It has been reported that simply leaving the graphite sample could result in the increase of Δ*E*_p_ due to the adsorption of either airborne or waterborne hydrocarbons.^53^ In the case of the highly concentrated KF, a protective barrier may form, which minimises the adsorption of organic species on the electrode surface, and stabilises the electrochemical reversibility of the redox complex. Indeed, early work on the voltammetry of this redox couple at graphite electrode showed that the voltammetry was sensitive to the identity of the supporting electrolyte cation, and adsorption of cation-based ion-pairs was invoked to explain this.^56^ The enhanced oxidation current at higher electrolyte concentrations, however, suggests that the additional current derives from oxidation of the adsorbate species, and that the oxidation kinetics are therefore more favourable at high electrolyte concentrations.

### Wettability of KF electrolytes

**Fig. 8:**
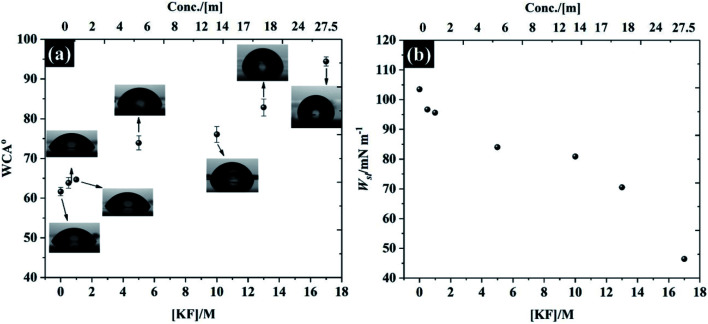
Wettability of freshly cleaved highly ordered pyrolytic graphite (a) water in air contact angle at *t* = 0 s, and (b) work of adhesion as a function of KF concentration.

In addition to the electrochemistry of the basal plane HOPG, the characterisation of the electrolytes' ability to wet the HOPG surface is demonstrated in Fig. 8. It is found that the freshly cleaved basal plane of HOPG exhibits relatively hydrophilic properties with a water in air contact angle (WCA) of 61.6° (±1.0°) for pure water, which agrees with previous observations.^57^ It is seen, however, that the WCA in Fig. 8a continually increases when the KF concentration was increased to 17 M: the WCA of 0.5 M and 17 M KF solutions are found to be 63.8° ± 1.3° to 94.3° ± 1.1°, respectively, which is due to the decrease of the liquid–air interface tension (*γ*_LG_, see Fig. S13† for the measurement of *γ*_LG_ at various KF concentrations). The expected pattern, for hydrophilic electrolytes, of an increase in *γ*_LG_ as the electrolyte concentration rises is not satisfied for KF, because of the positive surface potential of this salt.^57^ Typically, the surface potential of the electrolyte *e.g.*, NaCl, and KCl would decrease with increasing salt concentration.^58,59^ In order to relate the wettability with the electrolyte properties, the work of adhesion (*W*_sl_) *i.e.*, the reversible thermodynamic work required to isolate the interface of two phases at the equilibrium state (*e.g.*, graphite/water interface) to a separation distance of infinity,^60^ is presented in Fig. 8b. The energy required to separate the graphite/pure water interface is about 103.49 mN m^−1^. The reduction in contact angle is reflected in the lowering of *W*_sl_ on addition of more KF to the solution, the lowest *W*_sl_ is found to be 45.41 mN m^−1^ at 17 M KF. This relative hydrophobicity of the most concentrated electrolyte, 17 M KF, means the graphite surface can easily repel the water (at the interface), this prevents oxygen reduction and extends the potential window for electrochemical applications. This change of wettability with electrolyte concentration is general, as can be seen for the results of contact angle on hydrophilic PVDF membrane and cellulose paper supports, which can be found in Fig. S14 and S15.†

### Electrochemical performance of KF-based supercapacitors

**Fig. 9:**
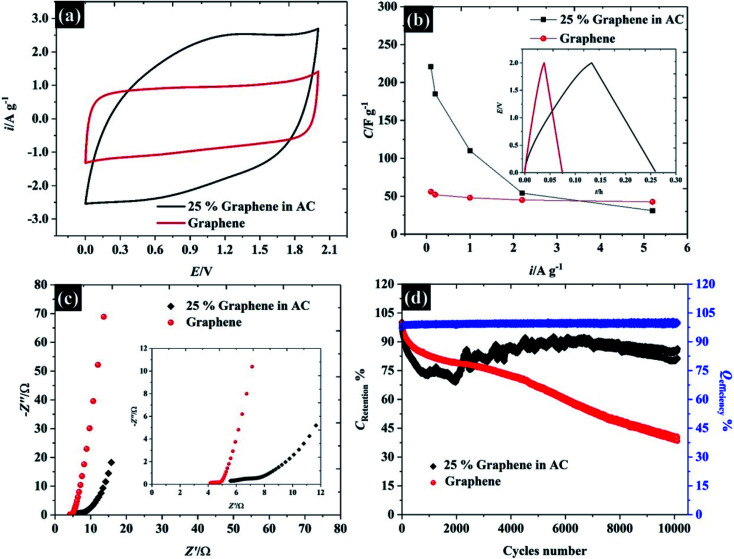
Performance of supercapacitor devices using 17 M KF electrolyte. (a) CV at 75 mV s^−1^, (b) capacitance and charge/discharge profile at 2.2 A g^−1^, (c) Nyquist plot, and (d) cyclic stability at 1.0 A g^−1^ over 10 000 cycles.

In order to demonstrate the applicability of these highly concentrated electrolytes from a macroscopic perspective, their use in symmetric supercapacitors has been explored as shown in Fig. 9. Herein, graphene and activated carbon have been used as representative carbonaceous electrode materials. By using highly concentrated KF (17 M, water-in-salt) electrolyte, both activated carbon and graphene exhibit an operating window potential of approximately 2.0 V (see Fig. 9a), which overcomes the thermodynamic limit of 1.23 V assumed to apply in aqueous solution, while the accessible potential window of the carbon electrode is usually reported to be less than 1 V.^1,61,62^ This is a proof of the advantages claimed for highly concentrated KF electrolytes from a macroscopic perspective for energy storage devices. The CV of those two materials reveals different charge storage mechanisms, the CV of graphene material displays a rectangular shape implying ideal capacitive behavior, where the charges adsorb only on the surface of the active materials,^1^ while the CV of activated carbon provides a more resistive shape due to the diffusion of ions into activated carbon pores.^61^ Meanwhile, the CV of activated carbon shows twice the specific current density of the graphene supercapacitor indicating a higher specific capacitance up to *ca.* 98 F g^−1^ (*cf.* specific capacitance of graphene is about 48 F g^−1^) at 75 mV s^−1^ (the calculation is based on total mass of the devices).

In addition to CV, the charge/discharge profile in the inset of Fig. 9b displays an excellent agreement with the CV: both activated carbon and graphene show linear responses, which reflects an ideal capacitive response for these devices, and the activated carbon exhibits a longer charge/discharge time than graphene.^63^ To gain more insight into the charge/discharge process, the applied current density of both devices was varied from 0.1 to 5.2 A g^−1^. The specific capacitance of activated carbon falls significantly from 221 to 31 F g^−1^ when the current density is increased to 5.2 A g^−1^, due to the limit of ion accessibility into the carbon pores.^64^ In contrast to activated carbon, the specific capacitance of the graphene supercapacitor is much less sensitive to the applied current (reducing from 56 to 42 F g^−1^), which confirms that the graphene supercapacitor stores most of the ions at the outer electrode surface. Note that the summary of the capacitive performance from previous publications in the topic of “water-in-salt electrolyte” is given in Table S6.† Herein, the water-in-KF demonstrates a comparable performance, in terms of available potential window and capacitance, to the water-in-LiTSFI^6,65–67^ and far better performance than concentrated NaClO_4_ (ref. 68–70) and LiNO_3_ electrolytes.^6^ Furthermore, the supercapacitor performances were tested by the AC impedance technique at the open circuit potential (OCP) as described in Fig. 9c.

The Nyquist plot of the graphene and activated carbon supercapacitor displays a straight line close to the vertical axis at the low frequency region confirming an ideal double-layer capacitor behaviour;^64^ however, the Nyquist plot at the high frequency region (inset Fig. 9c) of those two devices gave a different response. The electrolyte transport (Warburg diffusion term) plays an important role in the charge storage mechanisms of activated carbon supercapacitors while this element is relatively small for the graphene supercapacitor. This is again consistent with ionic diffusion in the graphene supercapacitor being much faster than in the activated carbon supercapacitor, due to the lower contribution of micropores to the overall capacitive response.^71^ Moreover, no semi-circle is found on both devices at the high frequency region and the solution resistance of both devices is nearly constant (about 4.1 Ω and 5.5 Ω for graphene, and activated carbon, respectively); thus, it can be concluded that the resistances of those two devices are dominated by the diffusion resistance, supporting the evidence from CV, charge/discharge, and impedance techniques. Finally, the cycle life stability of the as-fabricated supercapacitors was evaluated at 1.0 A g^−1^ over 10^4^ cycles as shown in Fig. 9d. Although the concentrated KF solution may be viewed as corrosive, the stable response obtained here suggests that the electrolyte is fully compatible with the cell components used. Both activated carbon and graphene supercapacitors exhibit an excellent coulombic efficiency, of approximately 99.5%; by contrast, the capacitance retention of the graphene supercapacitor drops down to 80% at about 1500 cycles and remains at 40% at cycle number 10 000. This may be due to the structural decomposition of graphene where the exfoliated material can restack, forming graphite-like structures.

In contrast to the activated carbon supercapacitor, the capacitance retention of activated carbon initially falls to ∼67% over first 2000 cycles. After 2000 cycles, the capacitance retention of activated carbon is then increased. This recovery may be due to air trapped inside the carbon pore;^72^ therefore, the electrolyte cannot fully utilise all the active area. Once the electrode is cycling, the electrolyte can penetrate further into carbon pores *via* an electrowetting effect.^73^ Eventually, the capacitance of the activated carbon supercapacitor remains constant at approximately 86% over 10 000 cycles indicating excellent cycle life time when used with the highly concentrated KF as water-in-salt electrolyte.

## Conclusions

In summary, this work introduces highly concentrated potassium fluoride as an aqueous electrolyte which is capable of delivering a wide working potential, in line with the “water-in-salt” concept recently exploited in energy storage devices. The electrochemistry, including the capacitance, from a microscopic perspective of these highly concentrated KF solutions was studied using highly ordered pyrolytic graphite (HOPG) as a proxy for carbonaceous materials. By using 17 M KF as an electrolyte and HOPG as a working electrode, the potential limits of the aqueous electrolyte could be overcome, achieving an accessible potential range of 2.6 V (assessed *via* the EIS technique). This phenomenon can be ascribed to at least three factors: (1) the lower solubility of dissolved oxygen as the electrolyte concentration increases, (2) the high viscosity of the electrolytes, which can reduce the electrochemical activity, (3) a lower electrolyte wettability, which can repel the water at the electrode surface, and indirectly induce a gain in accessible working potential. The capacitance of the basal plane graphite was also explored in each of the electrolyte concentrations to explain the capacitance of carbonaceous materials from a macroscopic view. Basal plane HOPG provides a capacitance *ca.* 3.5 to 3.8 μF cm^−2^ at the PZC and displays a stronger adsorption of potassium ions than of fluoride ions at higher electrolyte concentrations. Moreover, the faradaic electrochemistry of ferri/ferro cyanide were studied in order to describe the diffusion and heterogeneous electron transfer kinetics at the basal plane as well as the effect of the airborne/waterborne hydrocarbon adsorption. It is found that the use of 17 M KF can screen the carbon surface from contamination and enhance the electrochemical reversibility. Apart from the microscopic insights, we also demonstrated the applicability of these highly concentrated electrolytes (17 M KF) in symmetric supercapacitors using graphene and activated carbon electrode as “scalable” representatives of the carbon materials family. Activated carbon and graphene supercapacitors in 17 M KF exhibited a working voltage range of 2.0 V, with excellent capacitive and cyclic stability, suggesting that this cheap, inert electrolyte could be used successfully in commercial applications.

## Author contributions

P. I. performed all the experiments, except the supercapacitor tests (which were carried out by A. E). P. I. discussed the results, and wrote the manuscript, with R. D. The. All authors have given approval to the final version of the manuscript.

## Conflicts of interest

There are no conflicts to declare.

## Supplementary Material

SC-011-D0SC01754J-s001
