# Supplementary material for: Understanding the electrochemistry of “water-in-salt” electrolytes: basal plane highly ordered pyrolytic graphite as a model system
Source: Chem Sci. 2020 Jun 8;11(27):6978–89. doi: 10.1039/d0sc01754j (PMC8159404; doi:10.1039/d0sc01754j)
Supplement: SC-011-D0SC01754J-s001 [file SC-011-D0SC01754J-s001.pdf]

Electronic Supplementary Material (ESI) for Chemical Science.
This journal is © The Royal Society of Chemistry 2020

Supporting Information

Understanding the Electrochemistry of "Water-in-salt" Electrolytes: Basal Plane Highly Ordered Pyrolytic Graphite as a Model System

Pawin Iamprasertkun[a,b], *Andinet Ejigu*[a,b], *Robert A.W. Dryfe*[a,b,*]

[a]Department of Chemistry, School of Natural Sciences, University of Manchester, Oxford Road, Manchester M13 9PL, United Kingdom.

[b]National Graphene Institute, University of Manchester, Oxford Road, M13 9PL, United Kingdom.

KEYWORDS: Capacitance, Water-in-salt, Electrochemistry, Graphite, Potassium fluoride

EXPERIMENTAL SECTION

Chemicals and materials

Highly-ordered pyrolytic graphite (HOPG, grade ZYA, with a mosaic spread angle of 0.4° ± 0.1°) was purchased from Scanwel U.K. and used as the working electrode. Potassium chloride (≥ 99.0 %), Potassium fluoride (≥ 99.0 %), and Potassium ferricyanide (≥ 99.0 %) were purchased from Sigma-Aldrich. Hydrochloric acid (~37% with specific gravity = 1.18) was purchased from Fisher Scientific U.K. Limited. Agarose was purchased from Sigma-Aldrich (for reference electrode preparation). The aqueous electrolytes were prepared using ultra-pure water, of 18.2 MΩ cm resistivity, at 25 °C (Milli-Q Direct 8, Merck Millipore).

Preparation of Ag/AgCl reference electrode

Firstly, the silver wire (99.99% purity, Goodfellow Cambridge Limited: GF82496683, 0.20 mm diameter) was immersed into 0.5 M hydrochloric acid. The potential was then applied by chronoamperometry in steps from 0.5 V, 1.0 V, and 1.5 V, each for 30 minutes, using a two electrode configuration (platinum wire was used as a counter electrode).[1] The as-prepared Ag/AgCl electrode was then washed with *n*-propanol and de-ionized water several times to remove impurities and residual hydrochloric acid. To prepare an agarose gel, 3M KCl solution was heated to 80ºC. The agarose powder (3% wt.) was then added to 3 M KCl solution and stirred continuously until the gel dissolved. Next, the gel was carefully poured into a glass tube, to avoid the trapping of air bubbles. Then, the as-coated Ag/AgCl wire was put inside the glass body and filled with 3 M KCl electrolyte.[2] Lastly, the cap was sealed with the adhesive epoxy resin to protect against the evaporation of the electrolyte in the reference electrode.

Electrochemical measurements with the HOPG substrate

The electrochemical properties of the HOPG were measured using a potentiostat (PGSTAT302N, MetrohmAutolab) in a three-electrode configuration. Ag/AgCl and platinum wire were used as reference (RE) and counter electrodes (CE), respectively. HOPG, placed on a Si/SiO_2 substrate, was used as the working electrode (WE). The edge of the HOPG was covered with the silver epoxy (RS components, UK) and connected to a copper wire as can be seen in Figure S1. The HOPG surface to be exposed to the electrolyte was defined by a PTFE cell which had a small window of 3.0 mm diameter. Before electrochemical measurements, the platinum counter electrode was flame cleaned with a blue butane flame to remove impurities from the platinum surface. The HOPG was then cleaved using "scotch" tape to obtain a fresh basal plane HOPG. Subsequently, ~0.1 mL of electrolyte was then pipetted carefully into the PTFE cell. Note that this process was performed within 1 minute to avoid the adsorption of air-bound hydrocarbons on the basal plane HOPG.[3-5] Cyclic voltammetry (CV) was carried out in different electrolyte concentrations over a potential range extending from −1.6 V to 1.0 V vs. Ag/AgCl (for the study of the electrolyte concentrations) at a scan rate of 1 V s^{-1}. Furthermore, the capacitance-potential dependence was evaluated using electrochemical impedance spectroscopy (EIS) techniques by applying an AC voltage with 10 mV modulation over the frequency range from 100 kHz to 10 Hz at a fixed potential from −1.6 V to 1.0 V vs. Ag/AgCl (with 0.1 V increments). For the faradaic electrochemistry of $K_3Fe(CN)_6$, the CVs were performed through the same measurement procedures (over a potential range from −0.2 V to 0.8 V vs. Ag/AgCl), only the electrolyte consisted of 10 mM $K_3Fe(CN)_6$ and different KF concentrations (0.5, 1.0, 5.0, 10, 13, and 17 M) as supporting electrolytes. Note that the electrolytes were deoxygenated by purging nitrogen gas through the solution for 30 mins. before electrochemical measurement.

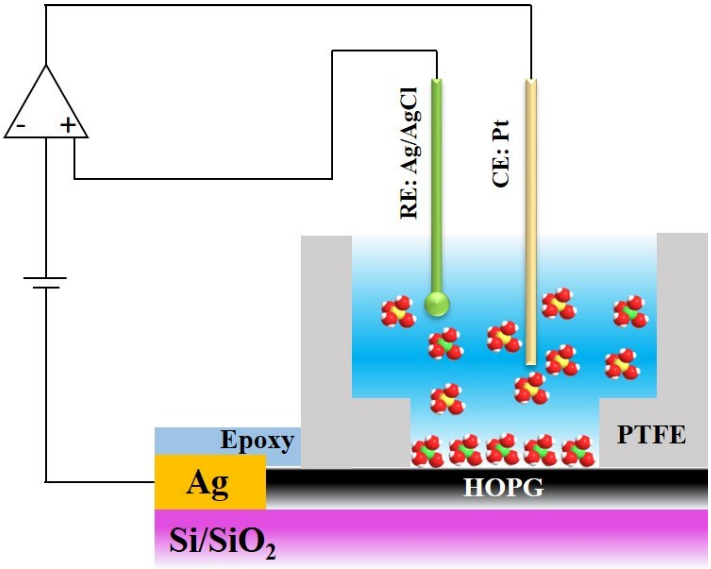

Figure S1. Schematic of the three electrode configuration used for capacitance measurement. HOPG (working electrode) is on a Si/SiO_2 substrate and connected *via* a silver epoxy. The PTFE cell defines the exposed area of HOPG (0.7068 cm^2).

Electrode preparation for supercapacitor tests

An electrode slurry was prepared by mixing 70 % of activated carbon (YEC-8B, Fuzhou Yihuan Carbon Co., Ltd), 25 % of graphene nanoplatelets (XGSciences, surface area 750 m^2/g) and carboxymethyl cellulose (CMC) binder (5% wt.%, Targray) in deionised water. The slurry was then coated using a compact doctor blade coater (MSK-AFA-III Casting Coater, MTI Corp) on a carbon-fixed Al foil with an Al_4C_3 (Toyal Carbo, Toyo aluminum K.K) substrate using doctor blading and dried for overnight at 80 °C. The cast electrodes were punched into small disks of 1.0 cm diameter for use in a CR2032 coin cell with 17 M KF as electrolyte. Note that the mass of the positive electrode was approximately twice that of the negative electrode.

Performance evaluation of supercapacitor

The performance of the supercapacitors were measured using a potentiostat (PGSTAT302N, MetrohmAutolab) in a two-electrode configuration. The CV of the as-fabricated supercapacitors was recorded at 75 mV s^{-1} between 0.0 v and 2.0 V, along with galvanostatic charge/discharge (GCD, from 0.1, 0.2, 1.0, 2.2, and 5.2 A g^{-1}, respectively). Electrochemical impedance spectroscopy was carried out at the open circuit potential (OCP) of the fabricated supercapacitor devices by applied 10 mV amplitude of sinusoidal frequency from 100 kHz to 0.01 Hz. Cyclic stability of the supercapacitors was evaluated by charge/discharge cycles at 1.0 A g^{-1} over 10000 cycles.

Capacitance calculation

To investigate the capacitance-potential curve of HOPG in different concentration of potassium fluoride electrolytes, the interfacial capacitance (C_A) of HOPG was calculated through the average capacitance at a frequency from 10 to 1000 Hz (when the phase angle approaches −90° this indicates capacitive behaviour) according to Eq. (S1)[6]:

$$C_A = \frac{-1}{2\pi f Z'' A} \quad \text{(S1)}$$

where f is the applied frequency (Hz), Z'' is the imaginary component of the impedance, and A is the exposed area of the HOPG. In addition to the capacitance from the fundamental perspective, the electrochemical properties of the supercapacitor using activated carbon and

graphene as active materials were investigated *via* CV, GCD, and EIS techniques as shown in Figure 9. The specific capacitances of the active materials were then calculated from GCD profiles by applying Eq. (S2)[6]:

$$C_s = 4\frac{i\Delta t}{m\Delta E} \quad \text{(S2)}$$

where i is the applied current, Δt is the discharge time from GCD profiles, m is the total mass, and ΔE is the working window potential of the supercapacitor.

Diffusion of the ferri/ferro cyanide redox species

In order to study the diffusion coefficient of the ferri/ferro ($Fe(CN)_6^{3-}/Fe(CN)_6^{4-}$) cyanide redox species, potential step chronoamperometry was carried out in 10 mM $K_3Fe(CN_6)$ using different KF concentrations (0.5 to 17 M) as supporting electrolytes at an initial potential of 0.0 V. The potential was stepped to 0.6 V vs. Ag/AgCl and held for 10 s (see Figure 4a). To calculate the diffusion coefficient, the chronoamperogram was then plotted as current *vs.* $t^{-1/2}$ as shown in inset Figure 4a. The diffusion coefficient of $Fe(CN)_6^{3-}/Fe(CN)_6^{4-}$ in each of the electrolyte concentrations is calculated from the gradient of the plot *via* Cottrell equation below[7]:

$$i = \frac{nFAc\sqrt{D}}{\sqrt{\pi t}} \quad \text{(S3)}$$

where n is number of electrons transferred per molecule, A is the exposed area of the electrode, F is the Faraday constant (96485 C mol^{-1}), and c is an initial concentration of the $K_3Fe(CN)_6$. To make a comparison, the diffusion coefficients were also calculated from the CV via the peak current (i_p) using the Randles-Sevcik equation in Eq. S4,[7].

$$i_p = 0.4463nFAc\left(\frac{nFvD}{RT}\right)^{1/2} \quad \text{(S4)}$$

where v is the scan rate, R is the molar gas constant (8.314 J mol^{-1} K^{-1}) and T is absolute temperature (298 K here),.

Heterogeneous electron transfer kinetics

To further describe the heterogeneous electron transfer kinetics of the $Fe(CN_6)^{3-}$ /$Fe(CN_6)^{4-}$ redox reaction, it is can be seen that the ΔE_p of the CV in Figure 3 and Figure S8 are between 80 and 292 mV [7], thus, the standard heterogeneous rate constant (k_0) was determined by method of Nicholson *via* the dimensionless kinetic parameter (ψ) defined in Eq. (S5).

$$\psi = k_0\left(\frac{D_O}{D_R}\right)^{\alpha/2}\sqrt{\frac{RT}{\pi nFvD_O}} \quad \text{(S5)}$$

where D_O and D_R are the diffusion coefficient of the oxidised and reduced components, respectively. α is the transfer coefficient. In this case, the D_O/D_R and α were assumed to be

1.20 and 0.5, respectively[7]. However, the Nicholson method only provides the ψ between the ΔE_p of 61 and 212 mV, which does not cover the complete range observed in this study; therefore, ψ was determined by the extended relationship of ψ as a function of ΔE_p as given in Eq. (S6)[8], which is an extension of the ψ function of Nicholson and Shain[9].

$$\psi = \frac{-0.6288 + 0.0021 n \Delta E_p}{1 - 0.017 n \Delta E_p} \tag{S6}$$

Viscosity measurement

The viscosity of the KF electrolyte solutions was measured by an Ubbelohde type viscometer (Paragon Scientific Ltd., serial number 31765) at 22 °C. Generally, the kinematic viscosity of each electrolyte was measured by flowing the liquid through the capillary. First, 20 mL of electrolytes were filled into the viscometer bulb. The liquid was then drawn upward to the mark level by vacuum. Then, the pressure was released and the time taken for liquid flow through the viscometer capillary (t) was measured. The kinematic viscosity (v) can be calculated using Eq. (S7) where C is the viscometer constant (0.002991 $mm^2\ s^{-2}$, calibration certificate number 31765/07/17).

$$v = Ct \tag{S7}$$

Electrochemistry on platinum microelectrodes

The electrochemistry of the platinum microdisk electrode of diameter 25 μm (CH Instruments, part number CHI108) was recorded with a potentiostat (PGSTAT302N, MetrohmAutolab) in a three-electrode configuration. The as-fabricated Ag/AgCl and platinum wire were used as reference (RE) and counter electrodes (CE), respectively. Before measure measurement, the platinum wire was flamed with the butane gas to remove the impurities on the platinum surface, the platinum microelectrode was polished with alumina lapping paper using diamond spray (particle size 1 μm) and the electrochemical cells were then setup in the Faraday cage. The CV response was recorded over potentials from 0.8 V to −0.2 V vs. Ag/AgCl in 10 mM $K_3Fe(CN_6)$ using KF as supporting electrolytes at scan rate of 100, 75, 50, 25 and 10 mV s^{-1}, respectively.

Water contact angle (WCA) measurement

The wetting properties of the concentrated electrolytes were investigated using a Theta Optical Tensiometer (Biolin Scientific, Finland) running OneAttension software (version 2.3). The measurement was performed through the sessile drop method on different substrates e.g., HOPG, hydrophilic PVDF membrane (pores diameter of 0.1 μm, Merck Millipore Ltd.), and 80 grams cellulose paper (supply by Banner). The droplet size in these experiments was controlled to a fixed volume of 1.5 μL. The measurements were carried out in a humidity chamber to minimise evaporation of the liquid. For the static electrolyte contact angle on the HOPG substrate, the images were recorded immediately ($t = 0$) after placing the electrolyte on top of the HOPG, the images were captured at a rate of 10.2 Hz. In order to measure the dynamic contact angle, the images were recorded at 1.9 Hz from t = 0 to t = 60 s.

Finally, the images were then analysed using the OneAttension software based on the Young-Laplace equation[10-11].

RESULTS SECTION

This work assesses the properties of class of aqueous electrolytes with wide operating potential known as “water-in-salts” using the KF salt; hence, KF solutions were prepared in ultra-pure water (Millipore, 18 MΩ cm resistivity) to yield concentrations of 0.5, 1.0, 5.0, 10, 13, and 17 M. These molarities correspond to molalities of 0.51, 1.03, 5.46, 12.34, 17.56, and 25.73 m, respectively. The mole fraction of these electrolytes was calculated as shown in Figure S2. The mole fraction of water has decreased linearly from ~1.00 to 0.68 as the amount of dissolved KF salt is increased, leading to the change in molar ratio of water: KF from 108.75 to 2.15 as shown in Table S1. It is obvious that, as the concentration of KF increased to 10 M (12.34 m), the electrolytes start to behave as a “water-in-salt” electrolyte due to the limited number of water molecules in the system.

Table S1: molar ratio of water: KF at different KF concentrations.

[M]	[m]	mol ratio
0.5	0.510378	108.7598
1	1.030282	53.87711
5	5.465923	10.1554
10	12.34468	4.496561
13	17.56094	3.160915
17	25.73384	2.157029

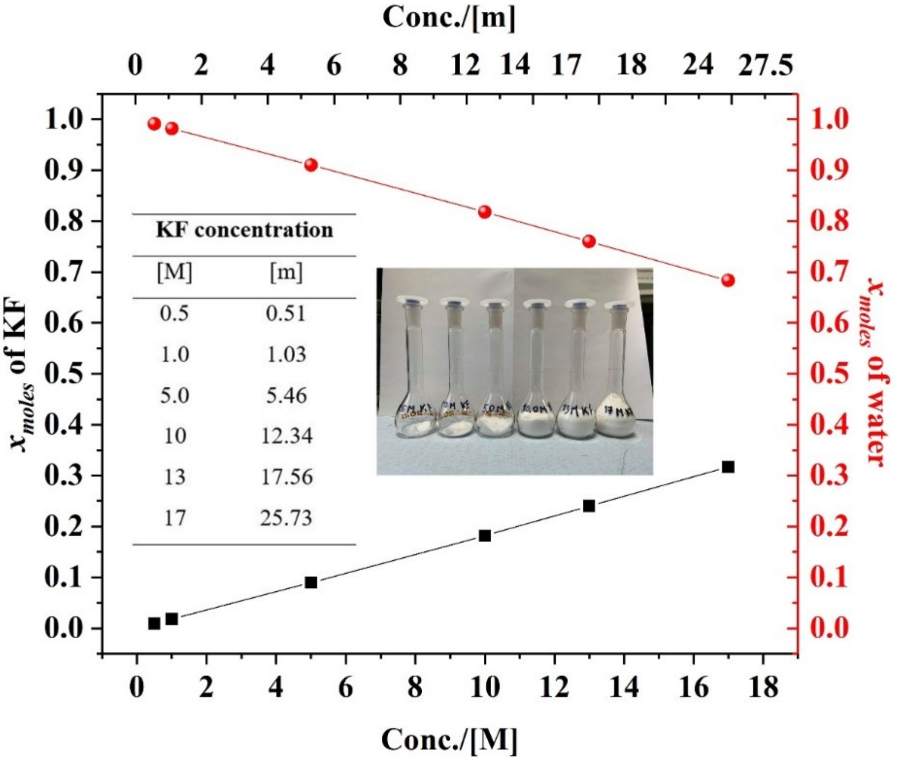

Figure S2: the mole fraction of the as-prepared potassium fluoride electrolyte at different concentrations.

In order to study the hydrogen evolution reaction at basal plane HOPG, the electrolytes were prepared by using different HCl concentrations from 1.0 to 5.0 mM in 0.1 M KCl (supporting electrolyte). The linear sweep voltammograms of the HOPG in those electrolytes are presented in Figure S3a. The onset potential of hydrogen evolution occurred about −0.7 V vs Ag/AgCl, when increasing the HCl concentration the reaction occurred at higher potentials[12]. It is evident that this reaction is diffusion controlled as the currents exhibit a linear dependence on the square root of scan rate ($v^{1/2}$) as can be seen in Figure S3b.

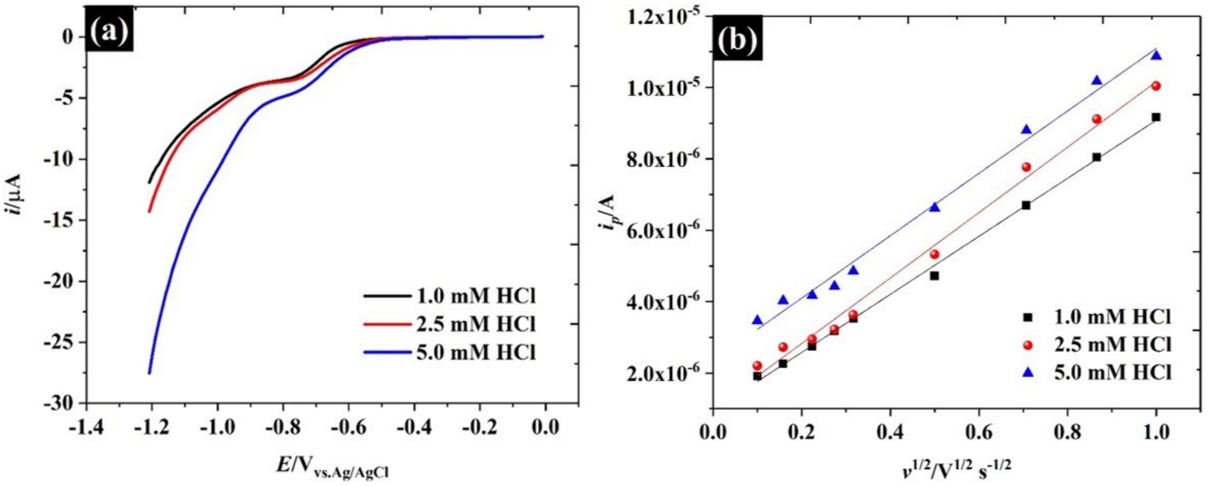

Figure S3: (a) LSV at 100 mV s^{-1}, and (b) i_p vs $v^{1/2}$ plot of basal plane HOPG in different hydrochloric acid concentrations in 0.1 M potassium chloride supporting electrolyte.

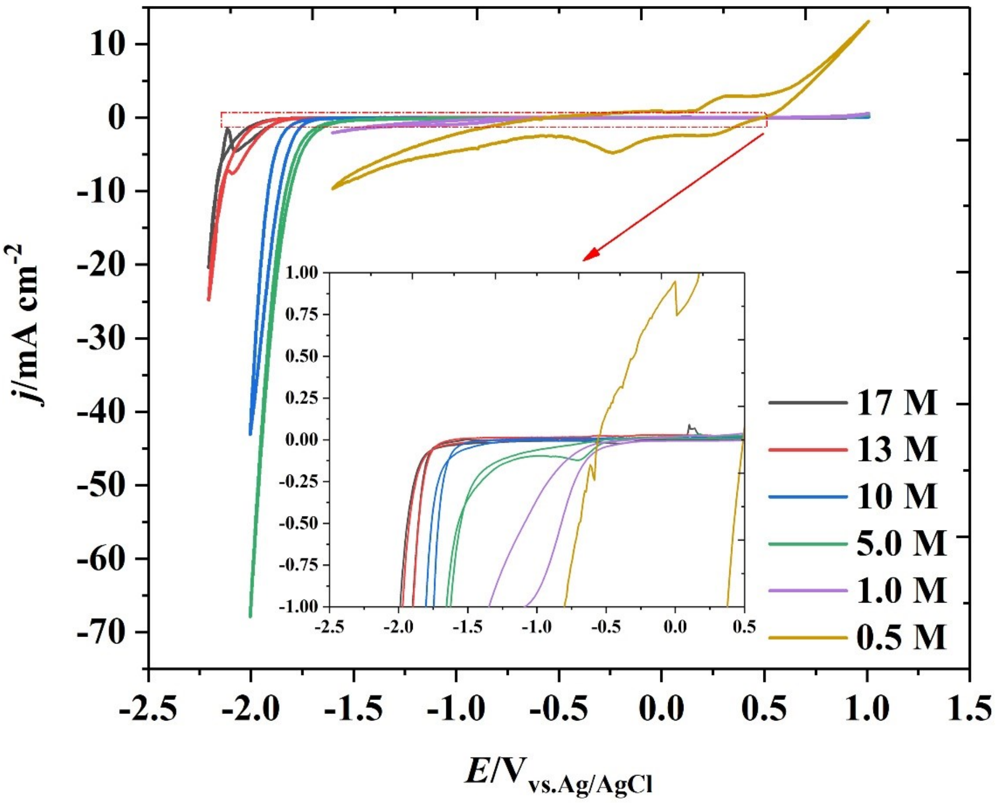

Figure S4: Extended CV of HOPG at 1.0 V s^{-1} at each KF concentration, with the vertical axis truncated at 1 mA cm^{-2} to highlight the dependence of potential window dependence on concentration.

In addition to the CV in Figure 1, the operating window potential of HOPG in 17 M KF was expanded to more positive values (up to 1.6 V). It is found that the faradaic reaction takes place when the potential exceeds 1.2 V vs. Ag/AgCl. This is because graphite oxidation occurs through anion intercalation[13], which can be clearly seen from the change in current after the potential exceeds 1.4 V. Once the potential reached 1.6 V, pronounced rough areas were seen to form on the exposed surface due to the exfoliation of basal plane HOPG. 17 M KF provides a negative potential limit of about −1.8 V vs. Ag/AgCl. It is obvious that the current increases significantly after the potential fell below −1.9 V. This faradaic current is associated with reduction of residual oxygen in the electrolyte, even though the electrolyte was degassed before the measurement. Note, the N_2 gas flowed above the PTFE cell during measurements in order to avoid dissolution of oxygen into the electrolyte. Thus, it can be concluded that the effective potential window of HOPG in 17 M KF is about 3.0 V; however, the operating potential window may be different due to the applied measurement conditions. Note that the voltammograms were recorded at a high scan rate because of an electrowetting effect that was seen at lower concentrations, where the initial contact angle is lower (*vide infra*[14]. The potential widow was also investigated *via* EIS to resolve its limits more precisely.

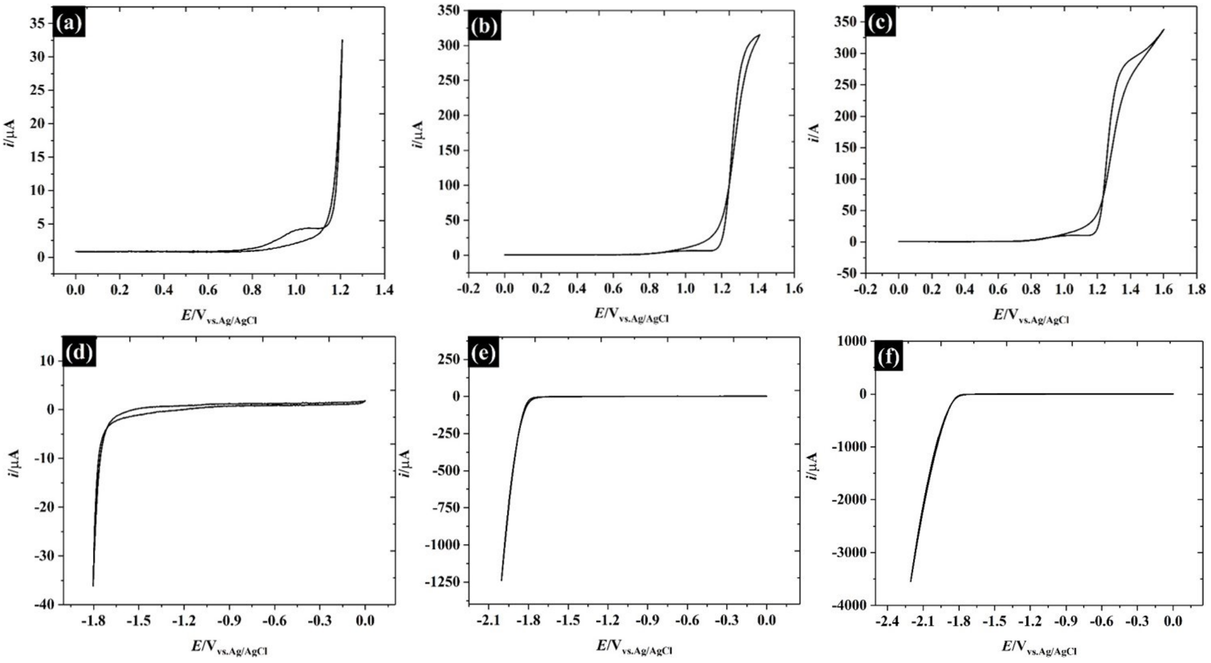

Figure S5: CV at 1 V s^{-1} of basal plane HOPG in 17 M KF at a window potential from (a) 0.0 to 1.2 V, (b) 0.0 to 1.4 V, and (c) 0.0 to 1.6 V, (d) −1.8 to 0.0 V, (e) −2.0 to 0.0 V, and −2.2 to 0.0 V vs Ag/AgCl.

EIS of the HOPG in each of the KF concentrations was performed from 100 kHz to 10 Hz in order to define the stable potential window of the as-prepared KF electrolytes as shown in Figure S6. One of the criteria is that the phase difference between current and potential should approach −90° if the system is displaying ideal capacitor properties[15]. Once the phase deviates from the ideal capacitor at that applied potential, then it will be deemed to have reached its potential limit. It can be seen that the stable potential limits of 0.5 M and 1.0 M KF are about −0.3 V to 0.6 V and −0.4 V to 0.6 V (vs. Ag/AgCl), respectively, which is typical of the range seen in a dilute aqueous electrolyte[16]. As the concentration of KF is

increased, the stable potential window becomes larger, as can be seen for the 5.0 M result. The working window potential of KF expanded to 1.2 V (from −0.6 to 0.6 V vs. Ag/AgCl), which co-incidentally corresponds to the thermodynamic limits of water stability[17]. Once the KF starts to behave as a water-in-salt electrolyte at 10 M, the Bode plot displays a wider potential window of up to 2.2 V (from −1.2 to 1.0 V vs. Ag/AgCl). One factor explaining the extension of the potential window seen with the most highly concentrated KF electrolytes (13 M and 17 M) is the lower O_2 solubility[18-19]. Specifically 13 M KF provides a wide potential window from −1.4 to 1.0 V (total of 2.4 V), while 17 M KF provides the potential window of up to 2.6 V (from −1.6 to 1.0 V vs. Ag/AgCl).

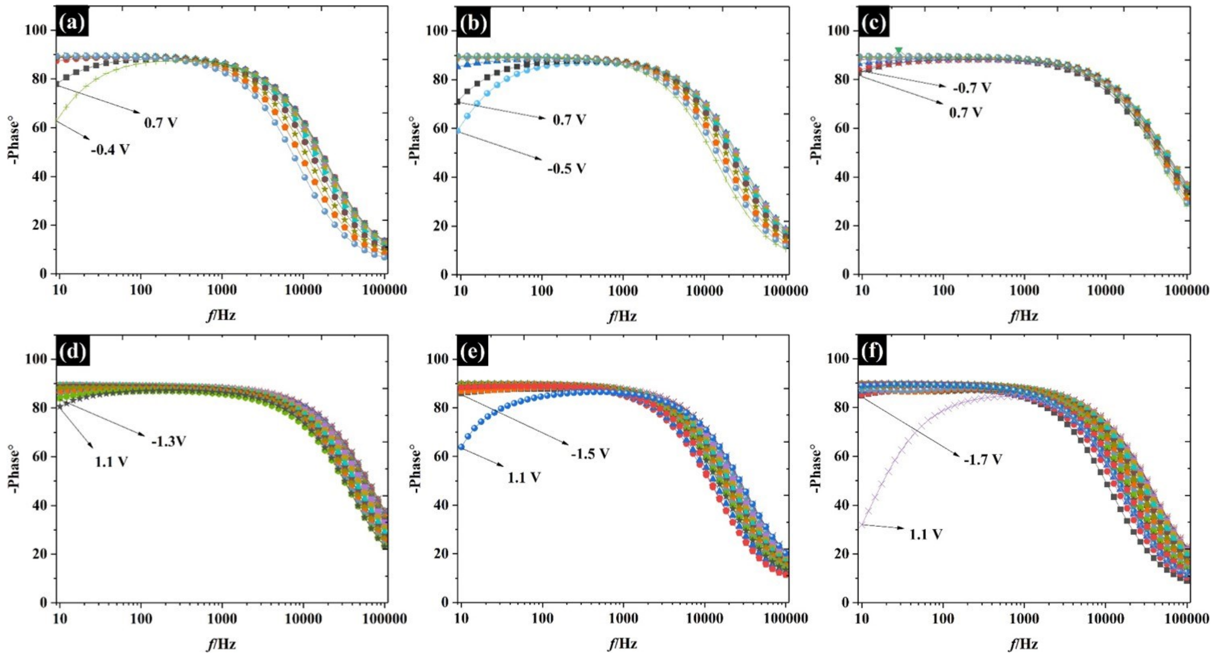

Figure S6: the Bode plots of the basal plane HOPG in different potassium fluoride concentrations (a) 0.5 M (0.51 m), (b) 1.0 M (1.03 m), (c) 5.0 M (5.46 m), (d) 10 M (12.34 m), (e) 13 M (17.56 m), and (f) 17 M (25.73 m).

Furthermore, we have presented the Nyquist plots of the HOPG in each of the KF concentrations as shown in Figure S7. It is clear that the Nyquist plots in Figure S7 are in excellent agreement with the Bode plots in Figure S6. At the stable potential of all electrolytes concentrations, the Nyquist plots exhibit a straight line close to the vertical axis indicating the ideal [RC] circuit of the capacitor[20]. Once again, when the potential exceeds the double layer region, the Nyquist plot deviates from the straight line due to the faradaic reaction. Hence, the capacitance derived from the EIS technique is calculated using Eq. (S1) as shown in Figure 2.

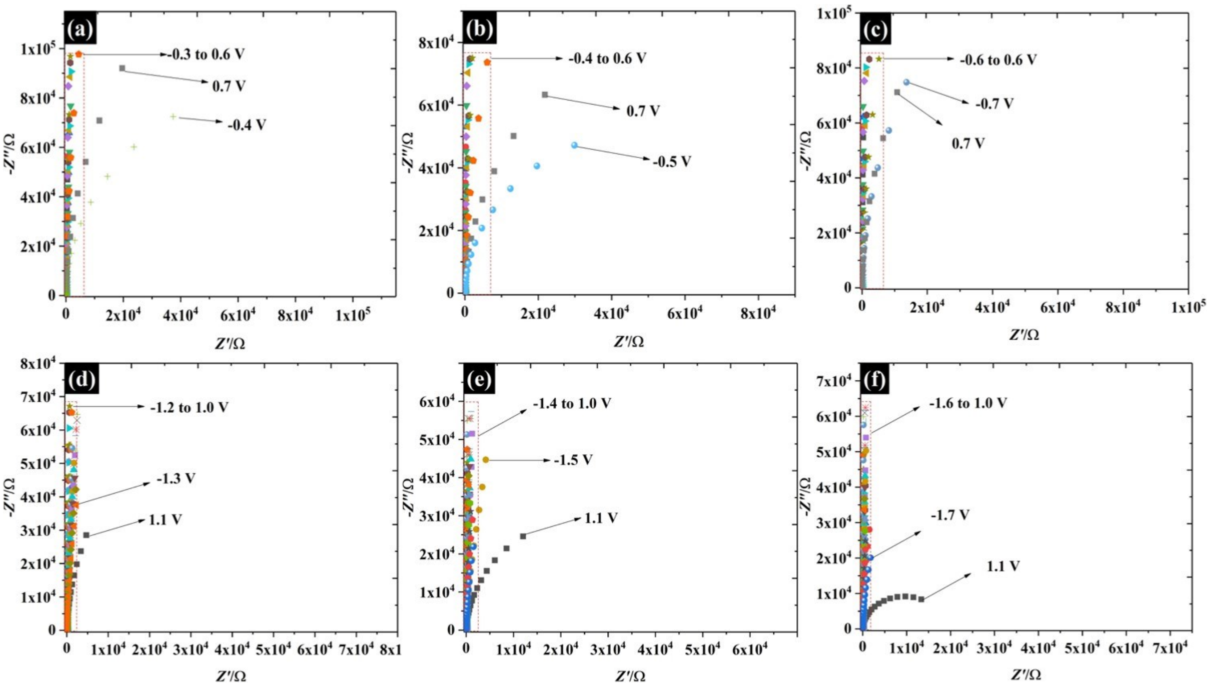

Figure S7: the Nyquist plot of the basal plane HOPG in different potassium fluoride concentrations (a) 0.5 M (0.51 m), (b) 1.0 M (1.03 m), (c) 5.0 M (5.46 m), (d) 10 M (12.34 m), (e) 13 M (17.56 m), and (f) 17 M (25.73 m).

Table S2: Summary of measured capacitance of basal plane HOPG in KF electrolytes.

E/V	Measured capacitance (*C*)/μF cm^{-2}					
	0.5 M	1.0 M	5.0 M	10 M	13 M	17 M
1.0					7.24	8.00
0.9					6.71	7.55
0.8					6.29	7.23
0.7				6.15	5.9	6.88
0.6	5.46	6.11	5.32	5.56	5.58	6.40
0.5	4.42	5.25	4.93	5.11	5.25	5.95
0.4	3.76	4.65	4.60	4.70	4.92	5.57
0.3	3.27	4.27	4.30	4.32	4.60	5.05
0.2	2.99	4.00	3.99	3.99	4.28	4.62
0.1	2.86	3.86	3.74	3.72	3.97	4.23
0	2.79	3.87	3.60	3.63	3.72	3.94
-0.1	2.69	3.91	3.60	3.58	3.61	3.81
-0.2	2.62	3.96	3.52	3.52	3.57	3.74
-0.3	2.59	4.04	3.55	3.49	3.49	3.64
-0.4		4.33	3.74	3.55	3.57	3.63
-0.5			3.85	3.71	3.82	3.79
-0.6			4.09	3.90	4.16	4.10
-0.7				4.14	4.36	4.47
-0.8				4.34	4.57	4.86
-0.9				4.57	4.87	5.25
-1				4.82	5.23	5.70
-1.1				5.13	5.66	6.20
-1.2				5.55	6.12	6.77
-1.3					6.71	7.40
-1.4					7.49	8.21
-1.5						9.10
-1.6						10.03

To further explain the faradaic electrochemistry of ferri/ferro-cyanide in KF electrolytes, the peak potential separations (ΔE_p) were evaluated from the CV (see Figure S8a). For a reversible, one-electron transfer reaction at room temperature, the separation between the oxidation and reduction peak potentials should be close to 59 mV and independent of scan rate[7]. For low electrolyte concentrations (0.5 to 5.0 M), it has been found that the ΔE_p are dependent on scan rate. The ΔE_p of 0.5 M KF at 10 mV s^{-1} is about 220 mV and it increased up to 292.5 mV at the highest scan rate (1 V s^{-1}) suggesting the process is a quasi-reversible electron transfer. As the concentrations were increased further to 1.0 and 5.0 M, respectively, the ΔE_p became lower, giving ΔE_p for 1.0 M KF of about 116.4 mV (10 mV s^{-1}) and 126.4 mV (1 V s^{-1}) while 5.0 M KF displays slightly lower ΔE_p about 100.6 mV at low scan rate (10 mV s^{-1}) and increased to 126 mV at the end; therefore, it can be concluded that the increase of KF concentration made the electron transfer processes more reversible. Interestingly, the ΔE_p of water-in-salt electrolytes (10, 13, and 17 M KF) display remarkably different responses, which are less dependent on scan rate and provide relatively low ΔE_p values of approximately 80 mV; however, the separations do not reach the ideal limiting value of 59 mV and, increased as the scan rate increased (for low KF concentration electrolytes), representing the effect of sluggish heterogeneous electron transfer kinetics[21]. Moreover, it is found that the ΔE_p at 10 mV s^{-1} of 13 and 17 M KF (Figure S8b) are significantly higher than those of faster scans. For these cases, the peak current ratio (i_{re}/i_{ox}) of 17 M KF is about 0.64 at 10 mV s^{-1}, and only approaches 1 at higher scan rates (~100 mV s^{-1}). This behaviour indicates that a further process accompanies the electron transfer process, most likely a catalytic chemical decomposition process which generates extra current on the reverse scan, (so-called EC' mechanism, see main text for further discussion)[21].

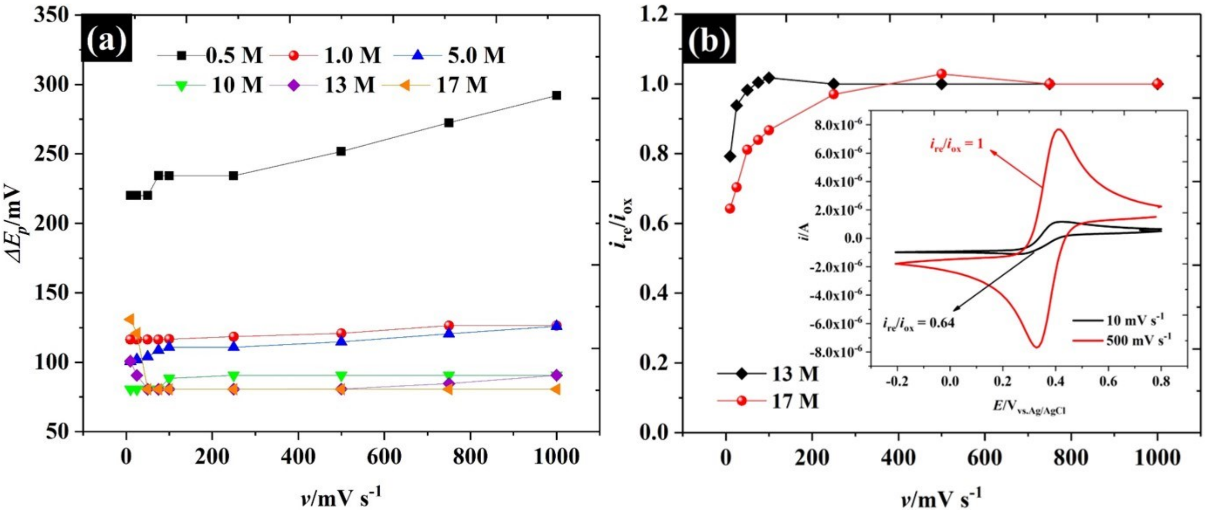

Figure S8: (a) ΔE_p as a function of scan rate for 10 mM potassium ferri/ferro cyanide in different potassium fluoride concentrations, and (b) the ratio between reduction and oxidation peak current with respect to scan rate for 13 and 17 M KF where the inset is the CV comparison between 10 mV s^{-1} and 500 mV s^{-1} of the maximum KF concentration at 17 M.

In addition to the Cottrell method, the diffusion coefficients were calculated using the Randles-Sevčik equation through the plots of peak current (i_p) as a function of square root of scan rate. It is found that the peak current for all electrolyte concentrations displays a linear dependence on the square root of scan rate indicating a diffusion controlled process. Hence, the diffusion coefficients of the ferri/ferrocyanide species were then determined from the gradient of the plots as shown in Figure S9. It can be seen that the diffusion coefficient from Randles-Sevčik equation is in reasonable agreement with the values from Cottrell equation: one reason for the deviation is that, strictly, the form of the Randles-Sevčik equation used applies to a reversible process. By applied Eq. (S5) and Eq. (S6), the standard electron transfer rate constants for the ferri/ferro species are obtained as shown in Table S3. The kinetic parameters calculated are in the same range and agree with previous reports[23].

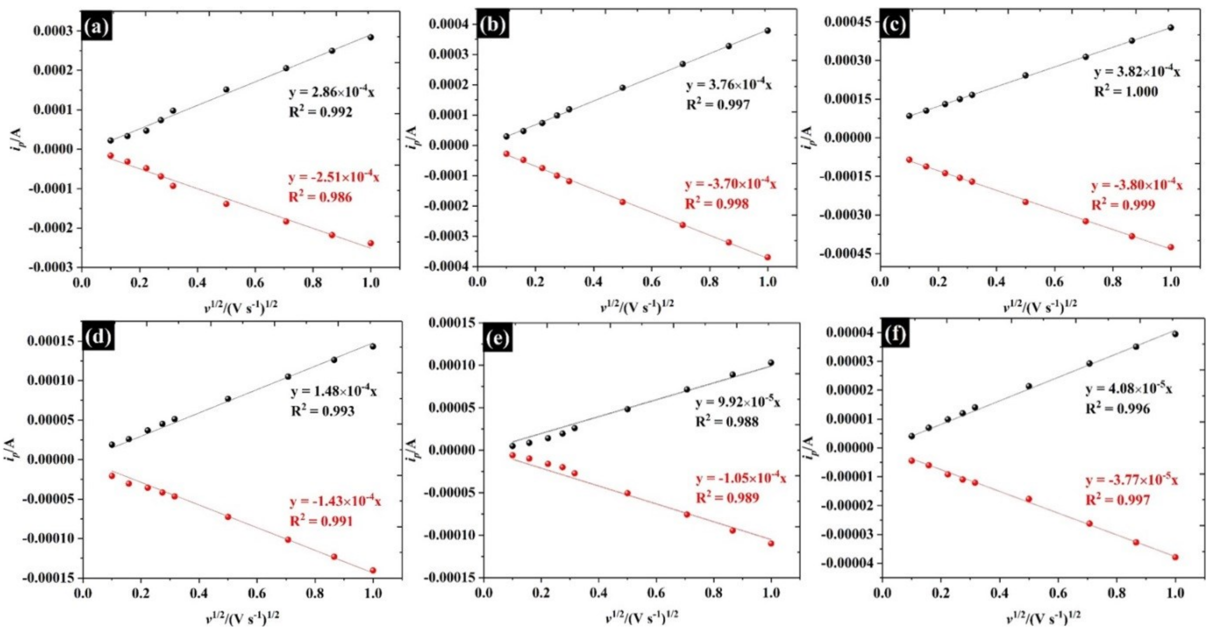

Figure S9: peak current as a function of square root of scan rates of HOPG in each of the electrolyte concentrations (a) 0.5 M, (b) 1.0 M, (c) 5.0 M, (d) 10 M, (e) 13 M, and (f) 17 M, respectively.

Table S3: Summary of the calculated diffusion coefficients and standard electron transfer rate constants from Cottrell and Randles-Sevčik equations.

Concentration/M	Cottrell equation		Randles-Sevčik equation	
	$D \times 10^{-6}$/cm^2 s^{-1}	$k_0 \times 10^{-3}$/cm s^{-1}	$D \times 10^{-6}$/cm^2 s^{-1}	$k_0 \times 10^{-3}$/cm s^{-1}
0.5	7.13	0.373	2.49	0.220
1.0	7.66	4.743	3.92	3.392
5.0	7.87	5.576	4.05	3.995
10	1.94	6.115	0.607	3.414
13	0.123	1.821	0.273	1.676
17	0.0124	0.646	0.0461	1.248

*k_0 are averages from all scan rate from 10 to 1000 mV s^{-1}

The viscosity of the electrolytes was measured by the Ubbelohde type viscometer through the kinematic viscosity (v), as shown in Figure S10. The kinematic viscosity is found to be 0.96, 0.98, 1.04, 1.54, 3.13, 4.29, and 6.45 cSt as the KF concentration increases from 0 to 17 M, respectively. The density of the electrolyte (ρ) was found to be 1008, 1028, 1205, 1291, 1495, and 1648 kg m^{-3} at KF concentrations ranging from 0.5 to 17 M, respectively. (The density of pure water at 25 °C was taken to be 997 kg m^{-3} to calculate the viscosity of pure water.)

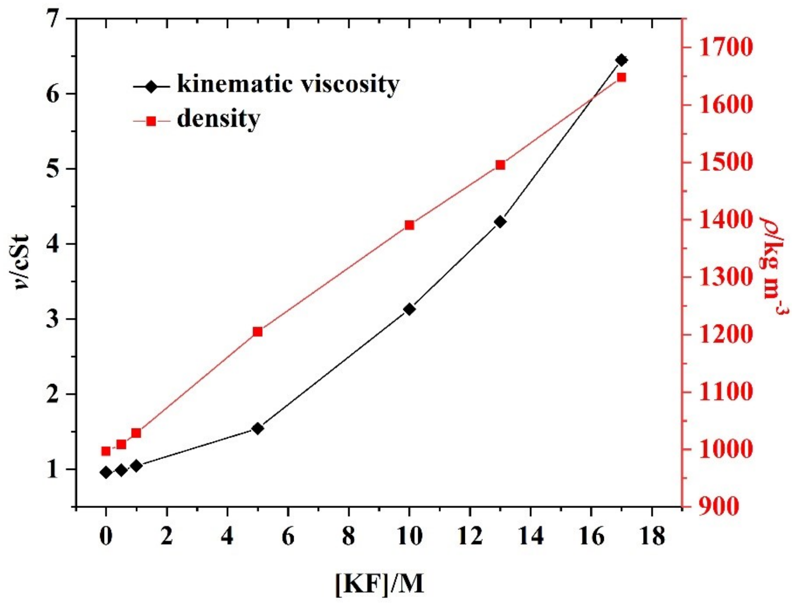

Figure S10: viscosity of potassium fluoride electrolyte from Ubbelohde type viscometer.

To further explain the viscosity of the prepared electrolytes, the Stokes-Einstein equation in Eq. (S8) has been applied to approximate the viscosity of fluids (η) from electrochemical techniques[21].

$$\eta = \frac{k_B T}{6\pi D r} \tag{S8}$$

where k_B is the Boltzmann constant (1.380649×10^{-23} J K^{-1}) and r is the hydrodynamic radius of $Fe(CN)_6^{4-}$ which is about 0.422 nm[24].

Table S4: Viscosity of the KF electrolyte from Ubblohde viscometry and Stokes-Einstein analysis of the electrochemical data.

KF concentration	Viscosity/η (cP)	
(M)	Ubblohde viscometer	Stokes-Einstein
0	0.96	-
0.5	1.00	0.72
1	1.08	0.68
5	1.86	0.66
10	4.36	2.66
13	6.43	42.01
17	10.63	418.00

In addition to the electrochemistry of ferri/ferro cyanide at the basal plane of HOPG, the CVs of the platinum microelectrode at different KF concentrations are shown in Figure S11. At the slowest scan rates, the microelectrode voltammetry tends to a steady state response due to the contribution of radial diffusion[7], although this is only asymptotically approached at the higher electrolyte concentrations due to the high viscosity of these solutions. It is found that the steady state current of the water-in-salt electrolyte (concentration above 10 M) was smaller by one order of magnitude than those of dilute electrolytes due to the diffusion of ferri/ferro cyanide species. Herein, the diffusion coefficient in each of the electrolyte concentration were calculated via Eq. S9[25].

$$i_{ss} = 4nFcDr_d \tag{S9}$$

where i_{ss} is the steady state current, and r_d is the radius of the microelectrode.

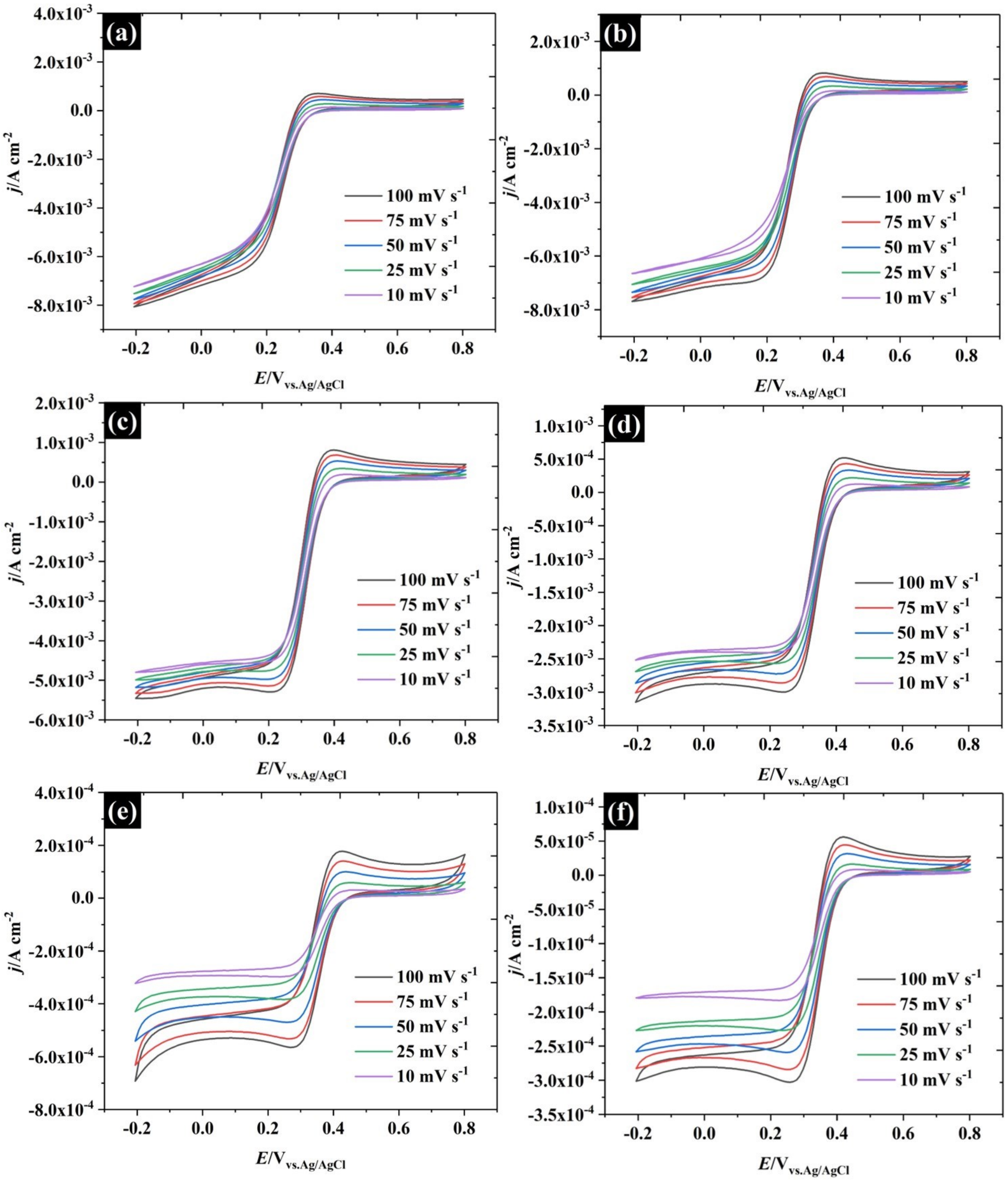

Figure S11: CV of platinum micro disk electrode (diameter of 25 μm) in each of the electrolyte concentrations (a) 0.5 M, (b) 1.0 M, (c) 5.0 M, (d) 10 M, (e) 13 M, and (f) 17 M, respectively.

Overall, it is evident that the diffusion coefficient of the ferri/ferro cyanide fell as the the KF concentration increased. This is consistent with the values obtained at the basal plane of macroscopic HOPG. At low KF concentrations, the platinum electrode yields diffusion coefficients of *ca.* 3×10^{-6} cm^2 s^{-1} (3.75×10^{-6}, 4.87×10^{-6}, 2.48×10^{-6} for 0.5, 1.0, 5.0 M, respectively), falling to 3.08×10^{-7} cm^2 s^{-1} at 10 M. The diffusion coefficient decreased further to 1.88×10^{-7}, 3.79×10^{-8} cm^2 s^{-1}, respectively, at 13 and 17 M as the electrolyte reached the water-in-salt regime. Moreover, the viscosity of the electrolytes was calculated using the Stokes-Einstein equation as shown in Figure S12.

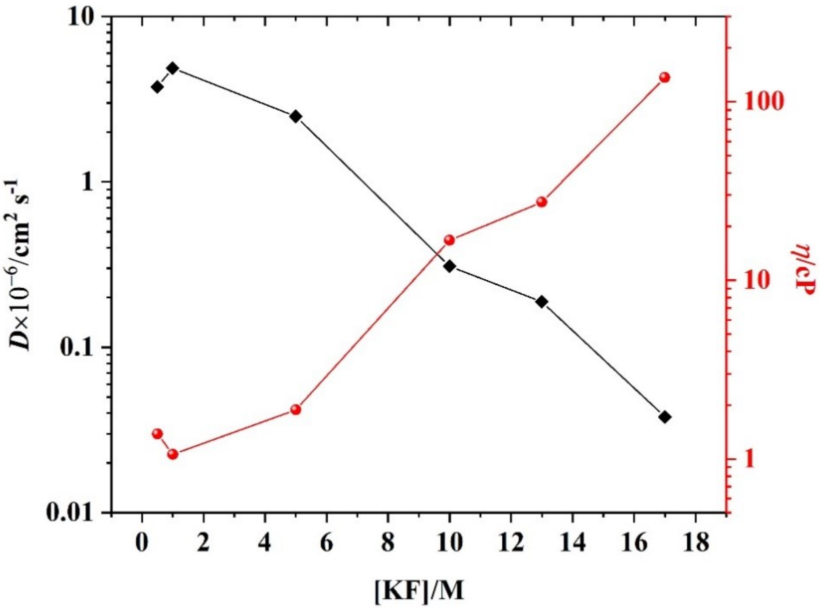

Figure S12: Diffusion coefficient of ferri/ferro cyanide and calculated viscosity (Stokes-Einstein) as a function of KF concentration determined using the Pt microdisk electrode.

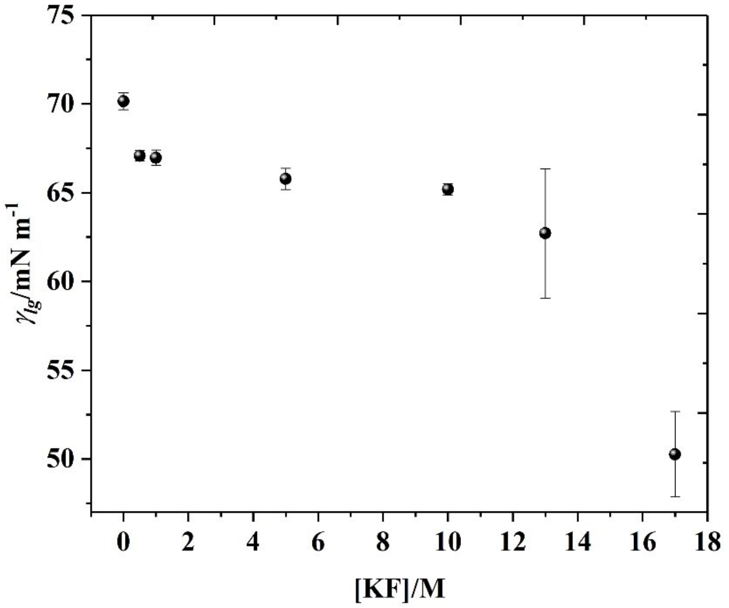

Figure S13: The interfacial tension of the water-air as a function of KF concentration.

To further describe the "work of adhesion (W_{sl})" in the main text, the work of adhesion is defined as the reversible work (from thermodynamic view point) at the equilibrium state, which is required to separate the interfaces of the two phases that are in contact. It can be calculated through the Young–Dupré equation by ignoring the solid-air interface tension as shown in Eq. (S10)[26].

$$W_{sl} = \gamma_{lv}(1 + \cos\theta) \quad \text{(S10)}$$

In order to explain the wettability of KF electrolytes on other substrates, the static and dynamic contact angle were measured on a hydrophilic PVDF membrane with a pore diameter of 0.1 μm (Figure S14). It can be clearly seen that the wettability of the KF suddenly changes from hydrophilic to hydrophobic when the KF concentration is increased to 5 M. It is found that droplets of the dilute electrolytes are not stable on the PVDF membrane due to the high wettability. The initial contact angle (t = 0) of pure water in air on the PVDF

membrane is about 50.4° and was found to decrease with time. The final reading was recorded at t = 8.69 s with the contact angle of 16.9°. This is due to the water being able to penetrate through the PVDF membrane[27]. As the electrolyte concentration was increased to 1.0 M, the work required to spread the droplet on the polymer surface increased slightly, yielding initial contact angles of 63.1° and 65.9°, respectively (for 0.5, and 1.0 M KF). The contact angle for those two electrolytes also fell with time and the final readings were recorded at t = 15.02 and 28.45 s, respectively. By contrast, the concentrated electrolytes (from 5.0 M) form a stable droplet and show non-wetting properties on the PVDF membrane. It is obvious that the initial contact angle of KF electrolytes grow from 98.5° (at 5.0 M) to 134.0° (at 17 M) suggesting that the cohesive force is greater than the adhesive force, which is in good agreement with the contact angle on the HOPG. Moreover, we have performed the contact angle measurement on the cellulose paper to make a comparison with the PVDF membrane as shown in Figure S15.

Table S5: Summary of water contact angle and work of adhesion on basal plane HOPG.

Concentration of KF/M	Water contact angle/°	Work of adhesion/mN m^{-1}
0 (pure water)	61.6 ± 1.0	103.4
0.5	63.8 ± 1.3	96.6
1.0	64.6 ± 0.3	95.6
5.0	73.9 ± 1.7	84.0
10	76.0 ± 1.9	80.8
13	82.8 ± 2.1	70.5
17	94.3 ± 1.1	16.4

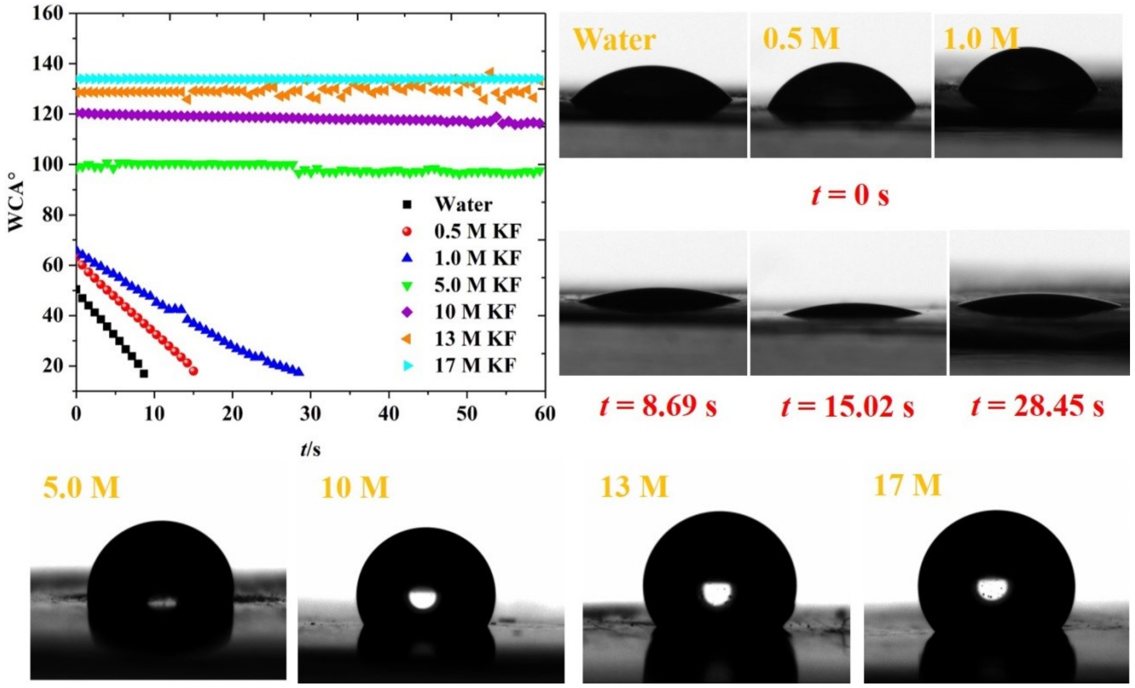

Figure S14: time dependence of water contact angle for potassium fluoride electrolyte solutions on hydrophilic PVDF membrane (pore diameter 0.1 μm).

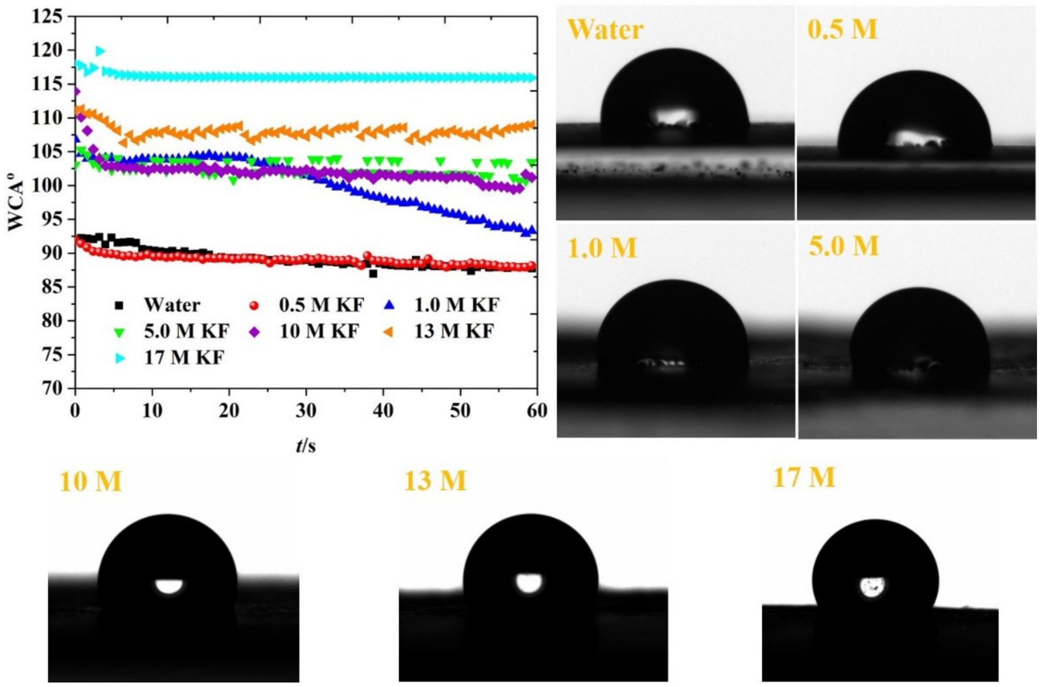

Figure S15: time dependence of water contact angle for potassium fluoride electrolytes on 80 grams m^{-2} cellulose paper.

Table S6: Electrochemical performance of various super concentrated (water-in-salt) electrolytes.

Salts	*c*	ΔE/V	materials	*C*/ F g^{-1}	methods	ref
$NaClO_4$	5 M	1.2	activated carbon	158	5 mV s^{-1}	[28]
	17 m	2.3	activated carbon	33	1.0 A g^{-1}	[29]
	17 m	2.5	activated carbon	31.9	1.0 A g^{-1}	[30]
LiTSFI	20 m	2.4	derived carbon	63	0.5 A g^{-1}	[31]
	5 M	2.5	activated carbon	183	5 mV s^{-1}	[32]
	5 M	1.4	MnO_2	239	2 mV s^{-1}	[33]
	21 m	2.2	carbon nanorod	44	1.0 A g^{-1}	[34]
HCOOK	40 M	3.4	activated carbon	275	10 mV s^{-1}	[35]
$LiNO_3$	5 M	1.0	MnO_2	224	2 mV s^{-1}	[33]
CH_3CO_2K	75% wt.	1.6	activated carbon	69	0.5 A g^{-1}	[34]
KF	17 M	2.0	activated carbon	221	0.1 A g^{-1}	*
	or	2.0	graphene	56	0.1 A g^{-1}	*
	25.7 m	2.6	HOPG	-	EIS	*
		3.0	HOPG	-	CV	*

*Super concentrated potassium fluoride (this work).

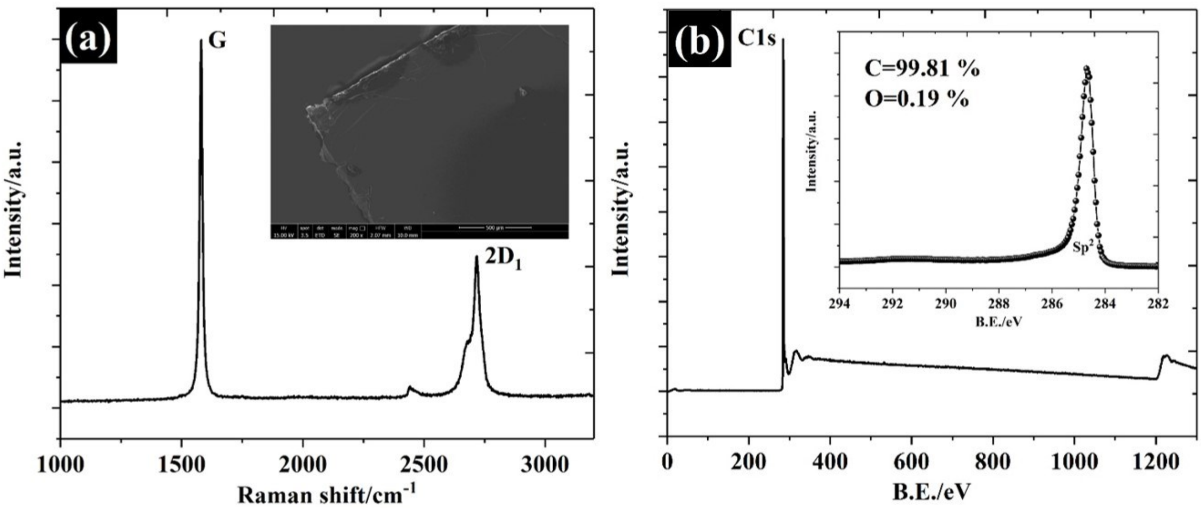

Figure S16: Characterisation of freshly cleaved HOPG sample (a) representative Raman spectrum, with a representative SEM image shown in the inset and (b) XPS survey spectrum where the inset shows the C1s spectrum.

REFERENCES

1. Iamprasertkun, P.; Hirunpinyopas, W.; Keerthi, A.; Wang, B.; Radha, B.; Bissett, M. A.; Dryfe, R. A. W., Capacitance of Basal Plane and Edge-Oriented Highly Ordered Pyrolytic Graphite: Specific Ion Effects. *The Journal of Physical Chemistry Letters* **2019,** *10* (3), 617-623.

2. Inamdar, S. N.; Bhat, M. A.; Haram, S. K., Construction of Ag/AgCl Reference Electrode from Used Felt-Tipped Pen Barrel for Undergraduate Laboratory. *Journal of Chemical Education* **2009,** *86* (3), 355.

3. Hurst, J. M.; Li, L.; Liu, H., Adventitious Hydrocarbons and the Graphite-water Interface. *Carbon* **2018,** *134*, 464-469.

4. Zou, Y.; Walton, A. S.; Kinloch, I. A.; Dryfe, R. A. W., Investigation of the Differential Capacitance of Highly Ordered Pyrolytic Graphite as a Model Material of Graphene. *Langmuir* **2016,** *32* (44), 11448-11455.

5. Kozbial, A.; Li, Z.; Sun, J.; Gong, X.; Zhou, F.; Wang, Y.; Xu, H.; Liu, H.; Li, L., Understanding the Intrinsic Water Wettability of Graphite. *Carbon* **2014,** *74*, 218-225.

6. Iamprasertkun P., Dryfe. R. A. W., The Capacitance of Graphene: From Model Systems to Large-Scale Devices. In *Nanocarbon Electrochemistry*, John Wiley & Sons: 2020; pp 33-84.

7. Bard, A.J.; Faulkner, L. R., *Electrochemical Methods: Fundamentals and Applications*. 2nd ed.; John Wiley & Sons: 2001; p 864.

8. Lavagnini, I.; Antiochia, R.; Magno, F., An Extended Method for the Practical Evaluation of the Standard Rate Constant from Cyclic Voltammetric Data. *Electroanalysis* **2004,** *16* (6), 505-506.

9. Nicholson, R. S., Theory and Application of Cyclic Voltammetry for Measurement of Electrode Reaction Kinetics. *Analytical Chemistry* **1965,** *37* (11), 1351-1355.

10. Cassie, A. B. D., Contact Angles. *Discussions of the Faraday Society* **1948,** *3* (0), 11-16.

11. Wenzel, R. N., RESISTANCE OF SOLID SURFACES TO WETTING BY WATER. *Industrial & Engineering Chemistry* **1936,** *28* (8), 988-994.

12. Ju, W.; Favaro, M.; Durante, C.; Perini, L.; Agnoli, S.; Schneider, O.; Stimming, U.; Granozzi, G., Pd Nanoparticles Deposited on Nitrogen-doped HOPG: New Insights Into the Pd-catalyzed Oxygen Reduction Reaction. *Electrochimica Acta* **2014,** *141*, 89-101.

13. Alliata, D.; Häring, P.; Haas, O.; Kötz, R.; Siegenthaler, H., Anion Intercalation Into Highly Oriented Pyrolytic Graphite Studied by Electrochemical Atomic Force Microscopy. *Electrochemistry Communications* **1999,** *1* (1), 5-9.

14. D. J. Lomax, P. Kant, A. T. Williams, H. V. Patten, Y. Zou, A. Juel and R. A. W. Dryfe, *Soft Matter*, 2016, **12**, 8798-8804.

15. de Levie, R., On Porous Electrodes in Electrolyte Solutions: I. Capacitance Effects. *Electrochimica Acta* **1963,** *8* (10), 751-780.

16. Borenstein, A.; Hanna, O.; Attias, R.; Luski, S.; Brousse, T.; Aurbach, D., Carbon-Based Composite Materials for Supercapacitor Electrodes: a review. *Journal of Materials Chemistry A* **2017,** *5* (25), 12653-12672.

17. Kanan, M. W.; Nocera, D. G., In Situ Formation of an Oxygen-Evolving Catalyst in Neutral Water Containing Phosphate and Co^{2+}. *Science* **2008,** *321* (5892), 1072.

18. MacArthur, C. G., Solubility of Oxygen in Salt Solutions and the Hydrates of These Salts. *The Journal of Physical Chemistry* **1916,** *20* (6), 495-502.

19. Suo, L.; Borodin, O.; Gao, T.; Olguin, M.; Ho, J.; Fan, X.; Luo, C.; Wang, C.; Xu, K., “Water-in-salt” Electrolyte Enables High-voltage Aqueous Lithium-ion Chemistries. *Science* **2015,** *350* (6263), 938.

20. Frackowiak, E.; Béguin, F., Carbon Materials for the Electrochemical Storage of Energy in Capacitors. *Carbon* **2001,** *39* (6), 937-950.

21. Lovelock, K. R. J.; Ejigu, A.; Loh, S. F.; Men, S.; Licence, P.; Walsh, D. A., On the Diffusion of Ferrocenemethanol in Room-temperature Ionic Liquids: An Electrochemical Study. *Physical Chemistry Chemical Physics* **2011,** *13* (21), 10155-10164.

22. Nekrassova, O.; Allen, G. D.; Lawrence, N. S.; Jiang, L.; Jones, T. G. J.; Compton, R. G., The Oxidation of Cysteine by Aqueous Ferricyanide: A Kinetic Study Using Boron Doped Diamond Electrode Voltammetry. *Electroanalysis* **2002,** *14* (21), 1464-1469.

23. Velický, M.; Bradley, D. F.; Cooper, A. J.; Hill, E. W.; Kinloch, I. A.; Mishchenko, A.; Novoselov, K. S.; Patten, H. V.; Toth, P. S.; Valota, A. T.; Worrall, S. D.; Dryfe, R. A. W., Electron Transfer Kinetics on Mono- and Multilayer Graphene. *ACS Nano* **2014,** *8* (10), 10089-10100.

24. Nightingale, E. R., Phenomenological Theory of Ion Solvation. Effective Radii of Hydrated Ions. *The Journal of Physical Chemistry* **1959,** *63* (9), 1381-1387.

25. Aoki, K., Theory of Ultramicroelectrodes. *Electroanalysis* **1993,** *5* (8), 627-639.

26. Ma, H.; Shen, Z.; Ben, S., Understanding the Exfoliation and Dispersion of MoS_2 Nanosheets in Pure Water. *Journal of Colloid and Interface Science* **2018,** *517*, 204-212.

27. Hirunpinyopas, W.; Prestat, E.; Worrall, S. D.; Haigh, S. J.; Dryfe, R. A. W.; Bissett, M. A., Desalination and Nanofiltration Through Functionalized Laminar MoS_2 Membranes. *ACS Nano* **2017,** *11* (11), 11082-11090.

28. Yin, J.; Zheng, C.; Qi, L.; Wang, H., Concentrated $NaClO_4$ Aqueous Solutions as Promising Electrolytes for Electric Double-layer Capacitors. *Journal of Power Sources* **2011,** *196* (8), 4080-4087.

29. Bu, X.; Su, L.; Dou, Q.; Lei, S.; Yan, X., A Low-cost "Water-in-salt" Electrolyte for a 2.3 V High-rate Carbon-based Supercapacitor. *Journal of Materials Chemistry A* **2019,** *7* (13), 7541-7547.

30. Dou, Q.; Lu, Y.; Su, L.; Zhang, X.; Lei, S.; Bu, X.; Liu, L.; Xiao, D.; Chen, J.; Shi, S.; Yan, X., A Sodium Perchlorate-based Hybrid Electrolyte with High Salt-to-water Molar Ratio for Safe 2.5 V Carbon-based Supercapacitor. *Energy Storage Materials* **2019,** *23*, 603-609.

31. Xu, S.-W.; Zhang, M.-C.; Zhang, G.-Q.; Liu, J.-H.; Liu, X.-Z.; Zhang, X.; Zhao, D.-D.; Xu, C.-L.; Zhao, Y.-Q., Temperature-dependent Performance of Carbon-based Supercapacitors with Water-in-salt Electrolyte. *Journal of Power Sources* **2019,** *441*, 227220.

32. Hasegawa, G.; Kanamori, K.; Kiyomura, T.; Kurata, H.; Abe, T.; Nakanishi, K., Hierarchically Porous Carbon Monoliths Comprising Ordered Mesoporous Nanorod Assemblies for High-Voltage Aqueous Supercapacitors. *Chemistry of Materials* **2016,** *28* (11), 3944-3950.

33. Gambou-Bosca, A.; Bélanger, D., Electrochemical Characterization of MnO_2-based Composite in the Presence of Salt-in-water and Water-in-salt Electrolytes as Electrode for Electrochemical Capacitors. *Journal of Power Sources* **2016,** *326*, 595-603.

34. Xiao, D.; Wu, Q.; Liu, X.; Dou, Q.; Liu, L.; Yang, B.; Yu, H., Aqueous Symmetric Supercapacitors with Carbon Nanorod Electrodes and Water-in-Salt Electrolyte. *ChemElectroChem* **2019,** *6* (2), 439-443.

35. Liu, T.; Tang, L.; Luo, H.; Cheng, S.; Liu, M., A Promising Water-in-salt Electrolyte for Aqueous based Electrochemical Energy Storage Cells with a Wide Potential Window: Highly Concentrated HCOOK. *Chemical Communications* **2019,** *55* (85), 12817-12820.

36. Tian, Z.; Deng, W.; Wang, X.; Liu, C.; Li, C.; Chen, J.; Xue, M.; Li, R.; Pan, F., Superconcentrated Aqueous Electrolyte to Enhance Energy Density for Advanced Supercapacitors. *Functional Materials Letters* **2017,** *10* (06), 1750081.
